# Prediction of reacting atoms for the major biotransformation reactions of organic xenobiotics

**DOI:** 10.1186/s13321-016-0183-x

**Published:** 2016-11-28

**Authors:** Anastasia V. Rudik, Alexander V. Dmitriev, Alexey A. Lagunin, Dmitry A. Filimonov, Vladimir V. Poroikov

**Affiliations:** 1 Laboratory for Structure-Function Based Drug Design, Institute of Biomedical Chemistry, 10/8 Pogodinskaya Str., Moscow, Russia 119121; 2 Medico-Biological Faculty, Pirogov Russian National Research Medical University, 1 Ostrovityanova Str., Moscow, Russia 117997

**Keywords:** Reacting atoms, Biotransformation, Drug metabolism, Site of metabolism, Xenobiotic, Prediction, PASS, LMNA descriptors, P450, SOM, SOMP, Aliphatic hydroxylation, Aromatic hydroxylation, N-glucuronidation, O-glucuronidation, N-oxidation, S-oxidation, C-oxidation, N-dealkylation, O-dealkylation

## Abstract

**Background:**

The knowledge of drug metabolite structures is essential at the early stage of drug discovery to understand the potential liabilities and risks connected with biotransformation. The determination of the site of a molecule at which a particular metabolic reaction occurs could be used as a starting point for metabolite identification. The prediction of the site of metabolism does not always correspond to the particular atom that is modified by the enzyme but rather is often associated with a group of atoms. To overcome this problem, we propose to operate with the term “reacting atom”, corresponding to a single atom in the substrate that is modified during the biotransformation reaction. The prediction of the reacting atom(s) in a molecule for the major classes of biotransformation reactions is necessary to generate drug metabolites.

**Results:**

Substrates of the major human cytochromes P450 and UDP-glucuronosyltransferases from the Biovia Metabolite database were divided into nine groups according to their reaction classes, which are aliphatic and aromatic hydroxylation, N- and O-glucuronidation, N-, S- and C-oxidation, and N- and O-dealkylation. Each training set consists of positive and negative examples of structures with one labelled atom. In the positive examples, the labelled atom is the reacting atom of a particular reaction that changed adjacency. Negative examples represent non-reacting atoms of a particular reaction. We used Labelled Multilevel Neighbourhoods of Atoms descriptors for the designation of reacting atoms. A Bayesian-like algorithm was applied to estimate the structure–activity relationships. The average invariant accuracy of prediction obtained in leave-one-out and 20-fold cross-validation procedures for five human isoforms of cytochrome P450 and all isoforms of UDP-glucuronosyltransferase varies from 0.86 to 0.99 (0.96 on average).

**Conclusions:**

We report that reacting atoms may be predicted with reasonable accuracy for the major classes of metabolic reactions—aliphatic and aromatic hydroxylation, N- and O-glucuronidation, N-, S- and C-oxidation, and N- and O-dealkylation. The proposed method is implemented as a freely available web service at http://www.way2drug.com/RA and may be used for the prediction of the most probable biotransformation reaction(s) and the appropriate reacting atoms in drug-like compounds.

**Graphical abstract:**
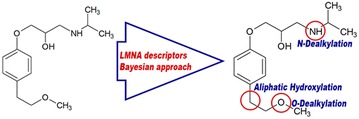
.

**Electronic supplementary material:**

The online version of this article (doi:10.1186/s13321-016-0183-x) contains supplementary material, which is available to authorized users.

## Background

Biotransformation is the biochemical modification of xenobiotics by living organisms that includes the involvement of specialized enzymatic systems. In the case of the biotransformation of active pharmaceutical ingredients, it is called “drug metabolism”. Drug metabolism influences the pharmacokinetics and therapeutic action of drug molecules [1] and may lead to the production of metabolites with significantly modified pharmacological and toxicological profiles, sometimes resulted to adverse effects of drugs. The pharmaceutical industry applies various in vitro and in vivo approaches at different stages of drug R&D to study the interactions of active pharmaceutical ingredients with drug-metabolizing enzymes, the metabolic fate of active pharmaceutical ingredients, and the structures and properties of potential metabolites. In contrast to “wet” experiments, computational (in silico) prediction of xenobiotic metabolites can be applied to virtual (not yet synthesized) compounds, enabling the optimization of the drug discovery process and generating a priori knowledge of metabolites that may be used for the creation of prodrugs. In silico methods may be applied in combination with various in vitro and in vivo models to optimize the metabolic stability and, in parallel, the target activity of compound series [2].

The site of metabolism (SOM) refers to the site of a molecule where a metabolic reaction occurs [3]. In many cases, SOMs are determined as atoms in a molecule that are modified by enzymes (mostly by P450s) [4]. In some works [5], the term SOM describes not only a single atom but also a group of atoms. There are various approaches to the prediction of SOMs for different CYPs. For example, MetaSite [6] is based on the combination of molecular interaction fields and molecular orbital calculations for the prediction of SOMs for various drug-metabolizing enzymes. The IDSite approach [7] is another example, which uses an induced-fit docking approach in combination with a quantum chemical model. SMARTCyp and RS-WebPredictor are two combined approaches for SOM prediction. SMARTCyp [8] uses a set of pre-calculated activation energies for molecular fragments in combination with topological descriptors, and RS-WebPredictor [9] uses pre-trained SVM models based on topological and quantum chemical descriptors and SMARTCyp reactivities. Tyzack et al. [10] showed that probabilistic classifiers implemented using randomly selected sub-classifiers on an ensemble basis with 2D topological circular fingerprints as descriptors can give reasonable SOM predictive performance. All the methods mentioned above are applicable for the site of metabolism prediction but do not estimate the structure of the metabolites. In some cases, for metabolic transformations catalysed by cytochromes P450, it is difficult to construct the structures of the metabolites based only on knowledge of the SOMs. The prediction of the SOM for aromatic and double-bonded carbons may imply the formation of different metabolites such as epoxides, alcohols, diols, and ketones. [11], while the prediction of the SOM for nitrogen atoms may imply the formation of N-oxides or dealkylated products [12].

The authors of SMARTCyp proposed to use the most common P450-catalyzed reactions to estimate which metabolite could be formed in the case of SOM prediction for various atoms and groups [11]. MetaPrint2D-React [13] provides associations of probable SOMs with the appropriate reactions. Zheng et al. [14] considered SOMs for six particular classes of P450-catalyzed reactions. A set of local quantum chemical properties were calculated with semi-empirical methods to represent the reactivity profile of a potential SOM. Quantum chemical calculations and feature selection procedure requires significant computational time.

As mentioned above, the term “SOM” sometimes means not a single atom but rather a group of atoms. In this work, we consider the particular reaction classes and introduce the term “reacting atom” that corresponds to a single atom. “Reacting atoms” is a term used in the representation of chemical reactions in computer programs—it is an atom that is present in both a reactant and a product and that changed adjacency [15].

For SOM determination the machine learning approaches should take into account the underlying mechanisms of enzymes’ action. But not always such information is available and results of SOM prediction can be interpreted correctly for understanding of structure of reactions products. For example, in many cases, researchers prefer to consider the carbon of the leaving group adjacent to the nitrogen as the SOM for N-dealkylation. This assumption is based on the hydrogen atom abstraction mechanism but does not take into account other possible one-electron transfer mechanisms of the N-dealkylation reaction [16]. We consider the nitrogen as the “reacting atom” in the case of the N-dealkylation reaction. Another problem with the uncertainty of the detection of the site of a molecule that is attacked by cytochromes P450 is associated with the mechanism of aromatic hydroxylation, which can be realized by the formation of an epoxide intermediate or by the “NIH shift”. Therefore, the direct determination of the SOM for the creation of training sets in machine learning approaches is problematic, and the interpretation of the predicted results is ambiguous.

The purpose of our study is to investigate the possibility of identifying the reacting atoms for the major classes of biotransformation reactions mediated by five human isoforms of cytochrome P450 and by all isoforms of the UDP-glucuronosyltransferase family.

In our approach we do not try to model or to mimic the hypothetical process of formation of intermediate compounds performed by P450. We use only the known information of the structures of the substrate and metabolite of the reactions for the creation of training sets to predict the reacting atoms of nine classes of reactions. We consider the classes of reactions of aliphatic and aromatic hydroxylation, N-, S- and C-oxidation, N- and O-dealkylation which, according to the Biovia Metabolite database [17], cover approximately 70% of all reactions catalysed by five major P450 isoenzymes (CYP1A2, CYP3A4, CYP2D6, CYP2C9, CYP2C19). In addition, we consider the N- and O-glucuronidation reactions, which cover almost all reactions that are catalysed by the UDP-glucuronosyltransferase family.

Using the term “reacting atom” and considering it as the site of a molecule of a substrate to which a particular structural fragment is added (or from which it is removed) allows one to identify the metabolite structures by the reacting atom prediction. Structural fragments that are added to the reactive atoms include hydroxyl (hydroxylation reactions), carbonyl or carboxyl (C-oxidation reactions), hydroxyl or oxo-group (N- and S-oxidation reactions), and glucuronyl (glucuronidation reactions) groups. In the case of dealkylation reactions, we considered the alkyl group as the fragment that is removed from the reacting atom represented by oxygen or by nitrogen.

Our method requires only structural formula of chemical compound and based on the analysis of “structure–reacting atom” relationships using a Bayesian approach and Labelled Multilevel Neighbourhoods of Atoms (LMNA) descriptors [18, 19]. It also does not take into account the spatial and stereochemical features of molecules of substrate and products.

## Results and discussion

### Identification of reacting atoms

We have selected biotransformations from the Biovia Metabolite database [17] that are catalysed by human CYP1A2, CYP2C19, CYP2C9, CYP2D6, and CYP3A4 and by all human UDP-glucuronosyltransferase isoforms and belong to nine reaction classes (aliphatic and aromatic hydroxylation, N- and O-glucuronidation, N-, S- and C-oxidation, and N- and O-dealkylation). These five cytochromes of P450s and UDP-glucuronosyltransferases metabolize the majority of drugs [20].

The reacting atoms were automatically identified in each substrate structure from the selected biotransformations. For automatically identification of reacting atoms, we are using APGL [21] and python-igraph [22] libraries. At first, all subisomorphisms between the substrate and product are found. Then algorithm check if the graph difference of the substrate and product structures is connected. If it is, then atoms with changed number of neighbor in isomorphic embedding are looking for. Examples of reacting atoms are shown in Table 1 (circled). Oxidation reactions are catalysed by cytochromes P450 and are mostly realized via heteroatom oxidation (N and S-oxidation) or carbon hydroxylation (aliphatic or aromatic hydroxylation). By aliphatic hydroxylation reaction, we mean a hydroxylation of the carbon atom that is not included in the aromatic rings. In the case of C-oxidation reactions, we consider the formation of carbonyl or carboxyl groups. N- and O-glucuronidation is catalysed by UDP-glucuronosyltransferases.

**Table 1:**
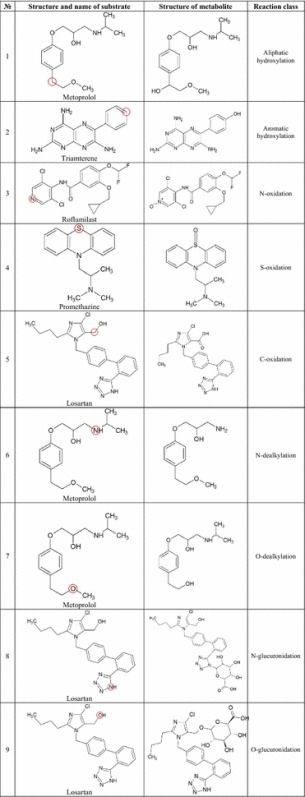
Examples of reacting atoms of the different types of biotransformation classes

### Training sets

The training sets were created by the generation of positive and negative examples represented by the structure with one labelled atom (SoLA) for each substrate from the selected set [18]. If a SoLA represents a chemical structure where a labelled atom is a known reacting atom, then this SoLA is considered a positive example. Otherwise, it is considered a negative example.

**Fig. 1:**
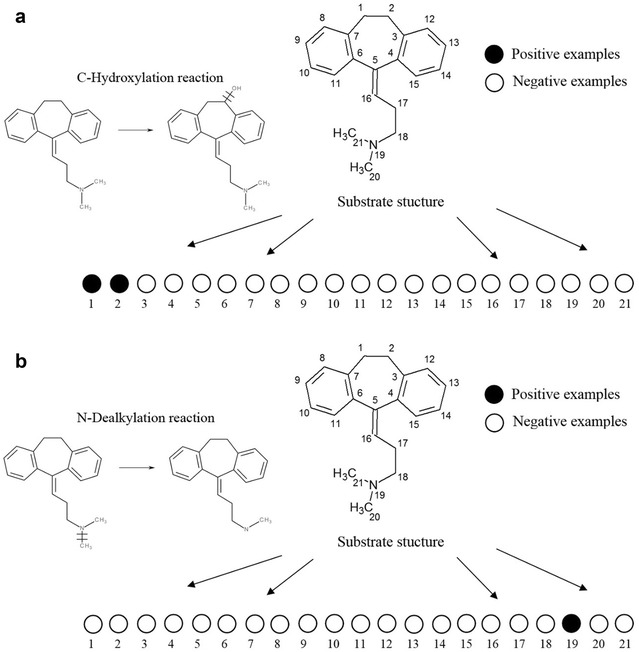
Schematic representation of SoLAs generated for amitriptyline. *Black circles* show positive examples of structures with known reacting atoms in case of **a** C-hydroxylation and **b** N-dealkylation reactions catalysed by CYP2D6. The *number* in the *upper string* indicates the atom number, which was labelled in the appropriate SoLA

For example (Fig. 1), 21 heavy (non-hydrogen) atoms of amitriptyline were labelled: one nitrogen and 20 carbon atoms. The interaction of amitriptyline with CYP2D6 leads to the appearance of two metabolites. Thus, SoLAs with the labelled substrate atoms No. 1 and 2 for C-hydroxylation and No 19 for N-dealkylation in the appropriate positions are considered to be positive examples in the appropriate training sets. In Fig. 1, all SoLAs represented as “circles” and numbers in the lower string indicate atoms that were labelled. SoLAs representing positive examples are shown as black circles, while those representing negative examples are shown as white circles.

Our training sets include substrates that are catalysed by five major cytochromes P450 and UDP-glucuronosyltransferases involved in drug metabolism via aliphatic hydroxylation, aromatic hydroxylation, N- and O-glucuronidation, N-oxidation, S-oxidation, C-oxidation, N-dealkylation and O-dealkylation reactions. We created separate training sets for each of the transformation types and for each of the reaction classes. We have used 4755 reactions of 3472 compounds. The total numbers of substrates, positive examples and two types of negative examples in the training sets are shown in the Table 2. The negative examples of the first type are the SoLAs, where labelled atom can be any heavy atom; the negative examples of the second type are the SoLAs, where labelled atom can be only the same chemical element as labelled atom in the positive examples. For instance, for S-oxidation the negative examples will be the SoLAs, where only sulphur atoms are labelled. The first type of preparing training set is more universal, but the second one better reflects the predictive power of the method.

**Table 2:** Characteristics of the training sets for prediction of reacting atoms and results of LOO cross-validation

Reaction classes	Substrates	Positive examples	Negative examples, 1st type	IAP, LOO CV, 1st type	Negative examples, 2nd type	IAP, LOO CV, 2nd type
Aliphatic hydroxylation	392	508	8575	0.91	6607	0.89
Aromatic hydroxylation	299	430	5890	0.92	4510	0.89
Hydroxylation	604	938	13,572	0.89	10,485	0.85
C-oxidation	69	69	1406	0.86	1062	0.85
N-oxidation	115	121	2405	0.99	241	0.78
S-oxidation	93	96	1947	0.99	7	0.99
N-glucuronidation	320	330	5611	0.99	509	0.86
O-glucuronidation	2264	2555	48,387	0.99	5645	0.93
N-dealkylation	401	422	8681	0.99	689	0.92
O-dealkylation	280	305	6095	0.99	675	0.85
Total	3472	4755	68,615		16,828	

The results of the training procedure and validation by LOO-CV for SAR models based on different training sets are also presented in Table 2. The invariant accuracy of prediction (IAP) criterion, similar to AUC (the area under the ROC curve) [23, 24], was used for the estimation of the accuracy of the created method. 20-fold cross-validation was also performed, and the same IAP values were obtained; therefore, they are not shown in Table 2.

As one may see from Table 2, the best accuracy is achieved for heteroatoms, which are easily distinguishable from the other atom types. However, the carbons that are the reacting atoms of aliphatic and aromatic hydroxylation are also predicted with reasonable accuracy, which suggests that one may use the method for the determination of reacting atoms. The accuracy of the reacting atom prediction for C-oxidation is lower than that in the other cases. This can be explained by the fact that the potential reacting atoms for C-oxidation and aliphatic hydroxylation could be the same if this atom is an aliphatic carbon atom without connected hydroxyl- or oxo-groups.

### Evaluation set

Drugs are usually inactivated by CYPs, but certain drugs are transformed to active substances. In these cases, the metabolites exhibit pharmacological activity and affinity to the target receptors of the pharmaceutical. The formation of active metabolites from the bioactivation of pharmacologically active drug substances is one of the issues of drug metabolism, and this is distinct from the case of prodrugs. For external validation, we used an evaluation set of 22 drugs that are transformed to active metabolites by various isoforms of cytochromes P450. The phenomenon of the changing of the therapeutic activity during the biotransformation is very important and often studied during the drug discovery process. The external evaluation set includes drugs belonging to various chemical classes from the publication of Obach [25].

**Fig. 2:**
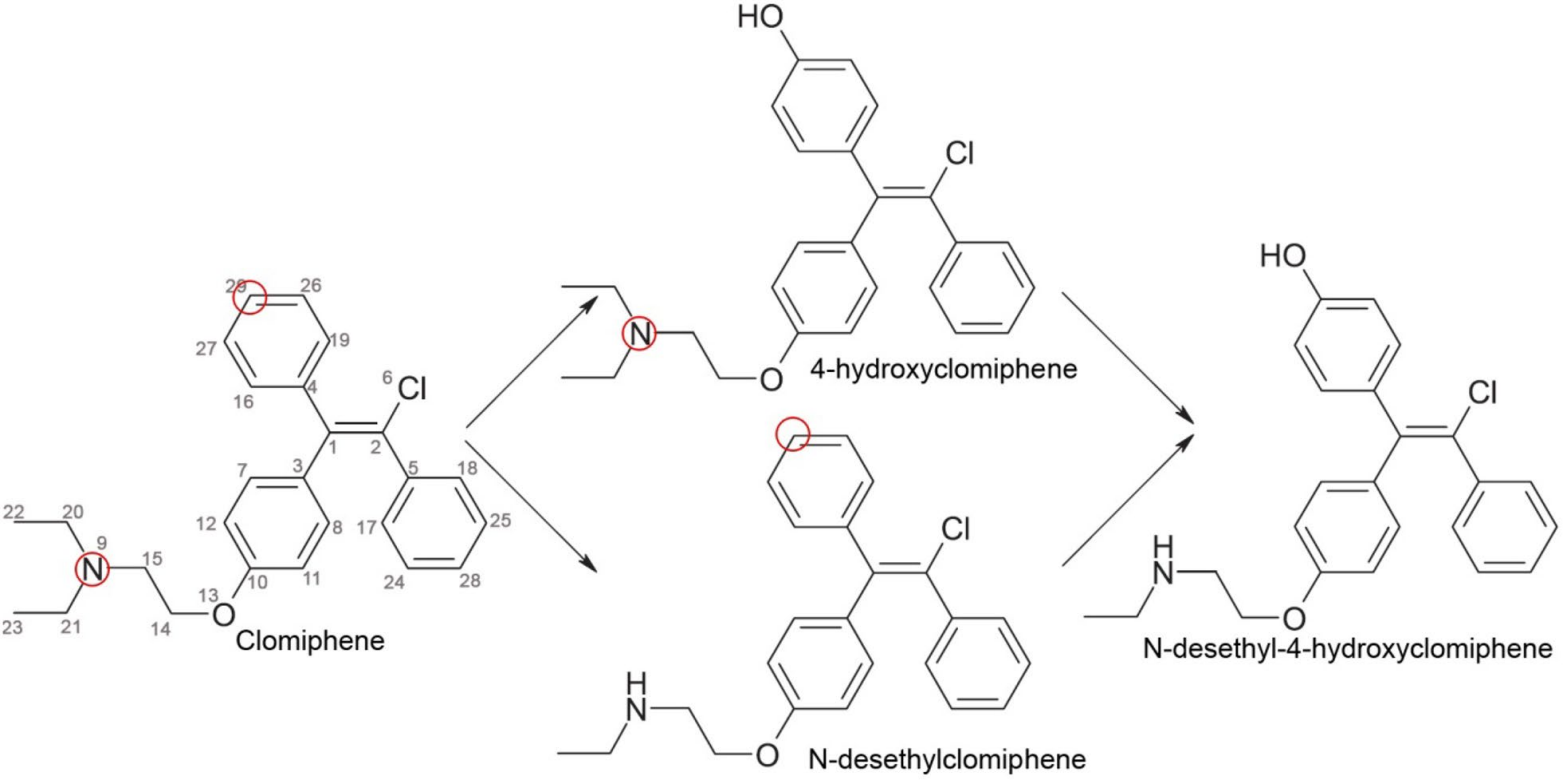
Biotransformation of clomiphene

These 22 compounds undergo reactions catalysed by five major P450 isoforms including aliphatic hydroxylation, aromatic hydroxylation, N-oxidation, C-oxidation, N-dealkylation and O-dealkylation. For example, for the clomiphene molecule (see Fig. 2) the aromatic hydroxylation at the para position of one of the phenyl rings catalysed by CYP2D6 (reacting atom is carbon No. 29) with the formation of 4-hydroxyclomiphene is known. Clomiphene also undergoes N-dealkylation (reacting atom is nitrogen No. 9) to form N-desethylclomiphene.

Because the publication of Obach [25] contains not all observed bioactivation reactions but only those with the formation of active metabolites, we enriched the evaluation set with the reactions presented in the Biovia Metabolite database [17] for these 22 compounds. The reactions from the Metabolite database were observed in both in vivo and in vitro experimental studies and catalysed by the five major P450 isoforms and by UDP-glucuronosyltransferases (we consider O- and N-glucuronidation reactions).

573 SoLAs were generated from the all compound structures presented in the evaluation set. The number of positive SoLAs depends on the reaction class and varies from four (in the case of C-oxidation) to 83 (in the case of “All reactions”). All these SoLAs, which are generated from the evaluation set, were excluded from the training sets, and then predictions were made for each of them. Training sets with the negative examples of the first type were used. The prediction results for every compound are presented in the Additional file 1.

We have also compared the prediction results obtained by our method with the prediction results provided by the MetaPrint2D-React (a web application/model “HUMAN”). To do this we prepared new training set “Hydroxylation” that consists of aliphatic and aromatic hydroxylation reaction together.

The prediction accuracy for the evaluation set is shown in Table 3, which contains four metrics for the estimation of the accuracy. Top-1, Top-2, Top-3 are metrics by which a molecule is considered to be correctly predicted if any experimental reacting atom is ranked as first, first or second, or first, second or third, respectively [26].

**Table 3:** Prediction results for the evaluation set

Reaction classes	Top-1	Top-2	Top-3	IAP
Aliphatic hydroxylation	0.83	0.92	0.92	0.95
Aromatic hydroxylation	0.64	0.91	1.00	0.94
Hydroxylation	0.82	0.94	0.94	0.93
Hydroxylation-MetaPrint2D-react	0.82	0.88	0.94	0.96
C-oxidation	1.00	1.00	1.00	1.00
N-oxidation	1.00	1.00	1.00	0.96
N-glucuronidation	1.00	1.00	1.00	0.99
O-glucuronidation	0.83	1.00	1.00	0.99
N-dealkylation	1.00	1.00	1.00	1.00
O-dealkylation	1.00	1.00	1.00	1.00

As one may see from Tables 2 and 3, the results of the internal and external validations show high accuracy in finding the reacting atoms for the considered biotransformation reactions.

As can be seen from the data in the Table 3, the estimates of prediction accuracy for Metaprint2D-React and for our method are comparable. Both methods require just only 2D structure of a molecule. The Metaprint2D-React method can predict the reacting atoms for more biotransformation reactions, then our method, but our method uses more specific names of reactions and may be used together with the preliminary prediction of biotransformation reactions.

### Web service for prediction of reacting atoms

The proposed method is realized in software that is freely available as a web service at http://www.way2drug.com/RA. It provides the prediction of the reacting atoms of aliphatic and aromatic hydroxylation, N- and O-glucuronidation, N-, S- and C-oxidation, and N- and O-dealkylation reactions.

The chemical structure could be uploaded using one of three different modes: drawing in Marvin [27], input as SMILES strings [28] or uploaded as a file in MDL (Biovia) Molfile format [29]. The prediction results display the structure with the numbered atoms and a table with the probable spectrum of the biotransformation reaction. This spectrum is calculated by PASS software [30] based on the SAR analysis of the training set containing more than 3500 substrates of cytochromes P450 and UDP-glucuronosyltransferases. The average accuracy of prediction in the LOO cross-validation (IAP) is 0.86. A detailed description of the training sets can be found at http://www.way2drug.com/ra/definition.php.

**Fig. 3:**
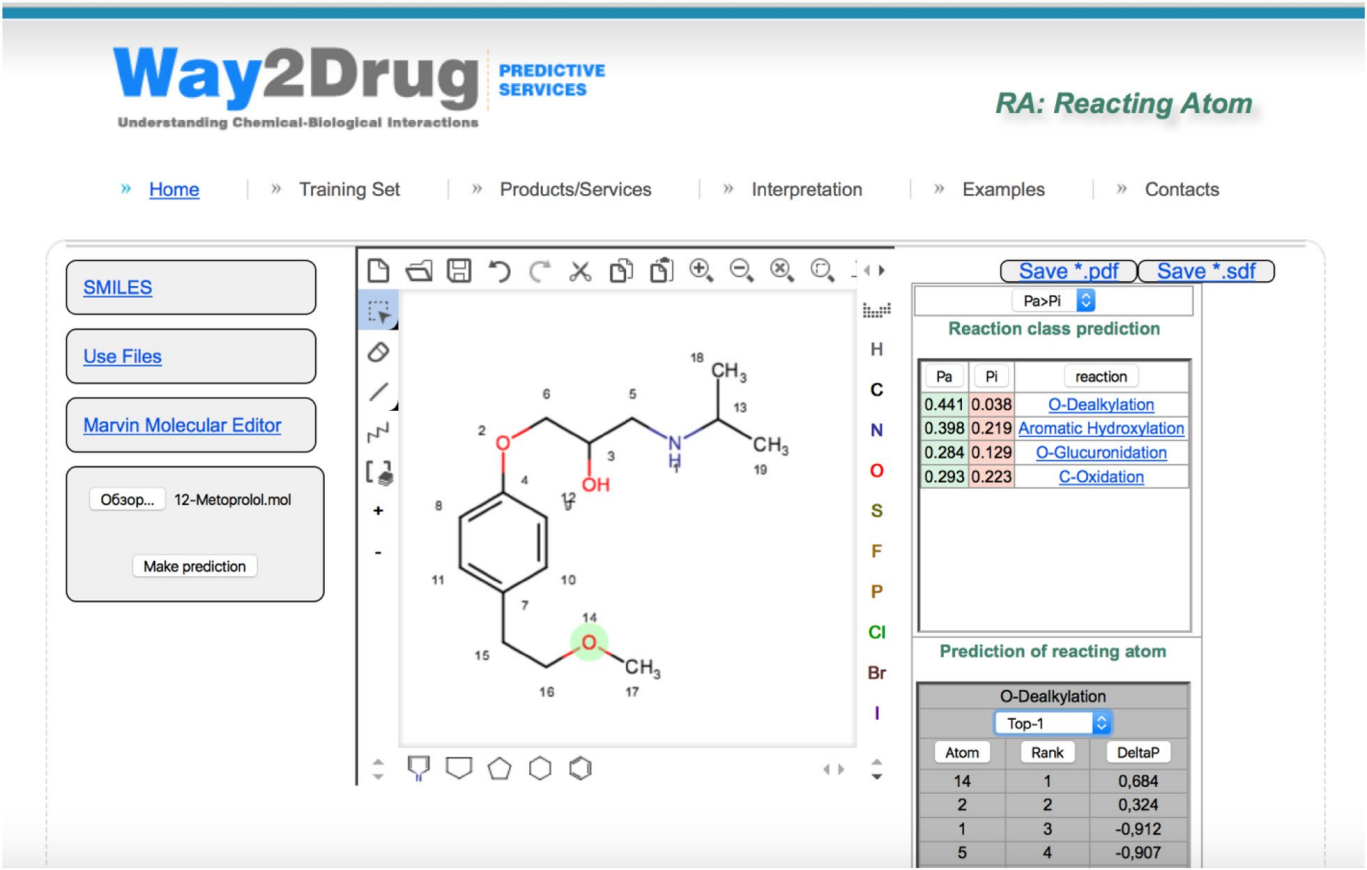
Example of prediction for metoprolol

By clicking on the reaction name, the user receives a table with the prediction of the reacting atoms of the selected reaction class. This table includes the atoms and their ranks according to the probability of being the reacting atoms of the selected reaction class. A drop-down menu with the top-metric is provided to the user. The atoms that correspond to the selected menu item are highlighted on the structure. An example of a prediction for Metoprolol is shown in Fig. 3.

The prediction results can be saved as *.sdf or *.pdf files. Web Server uses a MySQL server to store data, PHP and HTML code to implement the main interface. A Python script is used to produce independent sub-processes for generation input to the prediction program and data processing.

## Conclusions

Through interaction with different CYPs and with UDP-glucuronosyltransferases, xenobiotics may be transformed into metabolites by different reaction classes. We considered nine classes of reactions—aliphatic and aromatic hydroxylation, N- and O-glucuronidation, N-, S- and C-oxidation, and N- and O-dealkylation, for predicting the reacting atoms in the substrate.

In our approach, we use only the structures of the substrates for the prediction of the reacting atoms.

The leave-one-out training procedure and prediction for the external validation set, containing 22 drugs from Obach’s publication [25] and enriched by additional information from the Biovia Metabolite database, shows high accuracy (approximately 0.95 on average) for the prediction of the reacting atoms for each class of reaction.

The accuracy of the reacting atom prediction in the training procedure was higher (approximately 0.99) for the reaction classes involving heteroatoms (approximately 0.99). However, for the C-hydroxylation (aliphatic and aromatic) and C-oxidation reactions, the accuracy was also reasonable (approximately 0.89).

The proposed method is freely available as a web service at http://www.way2drug.com/RA/. On this site, a preliminary prediction of the reaction classes which, together with a combination of reacting class predictions, is equivalent to the prediction of the metabolite structures (because for each of the considered reactions, it is known which structural fragment is added to or removed from the reacting atom) is performed. The predicted structures of the metabolites can be used for the assessment of pharmacological and toxicological profiles and in mass spectrometry for the assessment of the positions where chemical fragments are added to or removed from the substrate structures.

## Methods

Each SoLA in a training set is described by a set of LMNA descriptors. Reaction class *T*_*k*_ could be one of eleven reaction classes (aliphatic and aromatic hydroxylation, N- and O-glucuronidation, N-, S- and C-oxidation, and N- and O-dealkylation reactions, “All reactions”, and “All CYP-mediated reactions”).

On the basis of SoLA representation by the set of *m* LMNA descriptors {*D*_1_, *D*_2_, …, *D*_*m*_}, the following values are calculated for reaction class *T*_*k*_

$$B_{k} = \frac{{S_{k} - S_{0k} }}{{1 - S_{k} S_{0k} }},$$

$$S_{k} = \text{Sin} \left[ {\frac{1}{m}\sum {Arc\text{Sin} \left( {2P\left( {T_{k} |D_{i} } \right) - 1} \right)} } \right],$$

$$S_{0k} = 2P\left( {T_{k} } \right) - 1$$

where *P*(*T*_*k*_) is the a priori probability that the labelled atom in SoLA is a reacting atom of reaction class *T*_*k*_ and *P*(*T*_*k*_ *|D*_*i*_) is a *conditional* probability that the labelled atom in SoLA is a reacting atom for reaction class *T*_*k*_ if descriptor *D*_*i*_ belongs to a set of LMNA descriptors of SoLA.

If *P*(*T*_*k*_ *|D*_*i*_) = 1 for all descriptors of SoLA, then *B*_*k*_ = 1. If *P*(*T*_*k*_ *|D*_*i*_) = 0 for all descriptors of SoLA, then *B*_*k*_ = −1. If there is no notable relationship between the descriptors of SoLA and the fact that the labelled atom in the SoLA is a reacting atom [i.e., *P*(*T*_*k*_ *|D*_*i*_) ≈ *P*(*T*_*k*_)], then *B*_*k*_ ≈ 0.

The simplest frequency estimations of the probabilities *P*(*T*_*k*_) and *P*(*T*_*k*_ *|D*_*i*_) are given by

$$P\left( {T_{k} } \right) = \frac{{N_{k} }}{N},\quad P\left( {T_{k} |D_{i} } \right) = \frac{{N_{ik} }}{{N_{i} }},$$

where *N* is the total number of SoLAs in the training set; *N*_*k*_ is the number of SoLAs in which the labelled atom is a reacting atom of reaction class *T*_*k*_; *N*_*i*_ is the number of SoLAs containing descriptor *D*_*i*_; and *N*_*ik*_ is the number of positive SoLAs (where the labelled atom is the reacting atom of reaction class *T*_*k*_) containing the descriptor *D*_*i*_.

During the training procedure, each SoLA is excluded from the training set, and a *B* value is calculated for it; so, the leave-one-out cross-validation (LOO CV) procedure is performed. Using the calculated *B* values for all SoLAs, the functions of the distribution of *B* values both for positive examples (*P*_*t*_(*B*)) and negative examples (*P*_*f*_(*B*)) are calculated.

During the prediction of the reacting atoms for a new compound, the set of all possible SoLAs with the appropriate LMNA descriptors is generated. The result is created on the basis of the prediction results of all SoLAs generated for the compound. Each SoLA relates to one appropriate potential reacting atom. The probabilities *P*_*t*_ and *P*_*f*_ are calculated for each SoLA of a new compound. *P*_*t*_ is the probability that a labelled atom in SoLA is a reacting atom of the appropriate reaction class, and *P*_*f*_ is the probability that a labelled atom in SoLA is not a reacting atom of the appropriate reaction class. The *deltaP* value is calculated as *deltaP* = *P*_*t*_ − *P*_*f*_.

Mathematically, the IAP value equals the probability that the estimation *deltaP* has the higher value for a randomly selected positive example (SoLAs in which the labelled atom is a reacting atom, *deltaP*_+_) than for a randomly selected negative example (SoLAs in which the labelled atom is not a reacting atom, *deltaP*_−_):

$${\text{IAP}} = Probability\left\{ {deltaP_{ + } > deltaP_{ - } } \right\}.$$

IAP is calculated as

$${\text{IAP}} = \frac{{NumOf\left\{ {deltaP_{ + } > deltaP_{ - } } \right\}}}{{N_{ + } \cdot N_{ - } }},$$

where *NumOf*{*deltaP*_+_ > *deltaP*_−_} is the number of cases where the *deltaP* for positive SoLAs exceeds the *deltaP* value for negative SoLAs. Thus, all pairs of SoLAs from the evaluation set are compared. *N*_+_ and *N*_−_ are the numbers of all positive examples and all negative examples in the set, respectively.

## Additional file

**Additional file 1.** The prediction results for compounds from evaluation set.

## Abbreviations

AUC: area under the ROC curve
IAP: invariant accuracy of prediction
LMNA: Labelled Multilevel Neighbourhoods of Atoms
LOO CV: leave-one-out cross-validation
PASS: prediction of activity spectra for substances
SOM: site of metabolism
SoLA: structure with one labelled atom
