# Supplementary material for: Prediction of reacting atoms for the major biotransformation reactions of organic xenobiotics
Source: J Cheminform. 2016 Nov 28;8:68. doi: 10.1186/s13321-016-0183-x (PMC5127045; doi:10.1186/s13321-016-0183-x)
Supplement: Supplementary file 1 — Additional file 1. The prediction results for compounds from evaluation set. [file 13321_2016_183_MOESM1_ESM.docx]

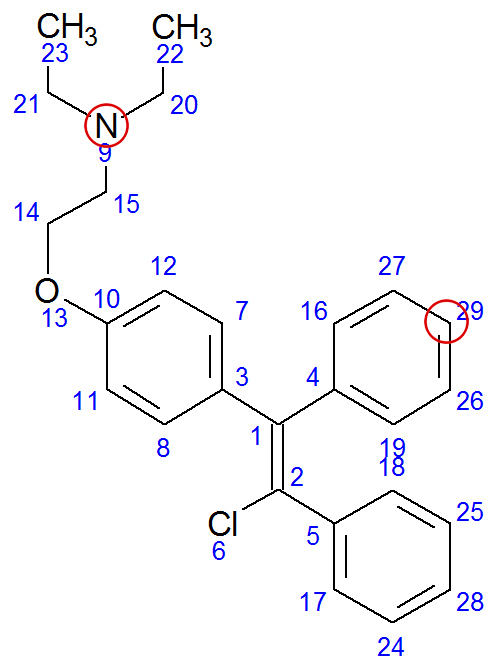


Figure 1. Structure of clomiphene

Table 1-1. Result of biotransformation class prediction for clomiphene

| Pa | Pi | Reaction |
| --- | --- | --- |
| 0.417 | 0.077 | O-Glucuronidation |
| 0.181 | 0.131 | N-Dealkylation |
| 0.126 | 0.361 | N-Oxidation |
| 0.081 | 0.444 | N-Glucuronidation |
| 0.092 | 0.562 | Aromatic Hydroxylation |
| 0.051 | 0.523 | O-Dealkylation |
| 0.080 | 0.794 | C-Oxidation |
| 0.020 | 0.830 | Aliphatic Hydroxylation |

Pa is the probability that this substance would undergo this reaction of biotransformation

Pi is the probability that this substance would not undergo this reaction of biotransformation.

The biotransformation reactions, that are experimentally observed, are highlighted

Table 1-2. Result of reacting atom prediction for clomiphene

| Atom number | Aliphatic  hydroxylation | Aromatic  hydroxylation | C-oxidation | N-oxidation | O-dealkylation | N-dealkylation | O-glucuronidation | N-glucuronidation |
| --- | --- | --- | --- | --- | --- | --- | --- | --- |
| 1 |  | 11(-0.280) |  |  |  | 3(-0.737) |  |  |
| 2 |  | 14(-0.484) |  |  |  | 6(-0.784) |  |  |
| 3 |  | 9(-0.235) |  |  |  | 12(-0.896) |  |  |
| 4 |  | 12(-0.308) |  |  |  | 9(-0.870) |  |  |
| 5 |  | 8(-0.148) |  |  |  | 4(-0.758) |  |  |
| 6 |  | 10(-0.262) |  |  |  | 2(-0.357) |  |  |
| 7 |  | 7(-0.003) |  |  |  | 15(-0.937) |  |  |
| 8 |  | 7(-0.003) |  |  |  | 15(-0.937) |  |  |
| 9 |  | 18(-0.964) |  |  |  | 1(0.918) |  |  |
| 10 |  | 13(-0.346) |  |  |  | 16(-0.947) |  |  |
| 11 |  | 4(0.249) |  |  |  | 19(-0.979) |  |  |
| 12 |  | 4(0.249) |  |  |  | 19(-0.979) |  |  |
| 13 |  | 19(-0.965) |  |  |  | 18(-0.965) |  |  |
| 14 |  | 17(-0.959) |  |  |  | 17(-0.950) |  |  |
| 15 |  | 18(-0.964) |  |  |  | 11(-0.894) |  |  |
| 16 |  | 6(0.029) |  |  |  | 14(-0.921) |  |  |
| 17 |  | 5(0.099) |  |  |  | 13(-0.900) |  |  |
| 18 |  | 5(0.099) |  |  |  | 13(-0.900) |  |  |
| 19 |  | 6(0.029) |  |  |  | 14(-0.921) |  |  |
| 20 |  | 16(-0.929) |  |  |  | 8(-0.856) |  |  |
| 21 |  | 16(-0.929) |  |  |  | 8(-0.856) |  |  |
| 22 |  | 15(-0.692) |  |  |  | 5(-0.779) |  |  |
| 23 |  | 15(-0.692) |  |  |  | 5(-0.779) |  |  |
| 24 |  | 2(0.405) |  |  |  | 10(-0.871) |  |  |
| 25 |  | 2(0.405) |  |  |  | 10(-0.871) |  |  |
| 26 |  | 3(0.316) |  |  |  | 13(-0.900) |  |  |
| 27 |  | 3(0.316) |  |  |  | 13(-0.900) |  |  |
| 28 |  | 1(0.478) |  |  |  | 7(-0.831) |  |  |
| 29 |  | 1(0.478) |  |  |  | 7(-0.831) |  |  |

The rank of the prediction is presented in the column.

DeltaP = Pa-Pi is presented in the brackets; Pa is the probability that this atom would be the reacting atom and Pi is the probability that it would not be the reacting atom of the specified reaction of biotransformation.

The real reacting atoms are highlighted.


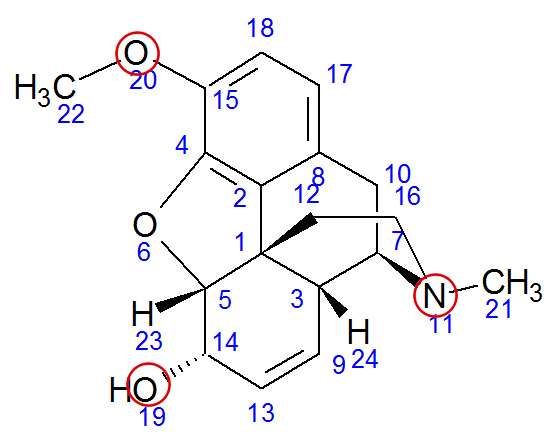


Figure 2. Structure of codeine

Table 2-1. Result of biotransformation class prediction for codeine

| Pa | Pi | Reaction |
| --- | --- | --- |
| 0.625 | 0.028 | O-Glucuronidation |
| 0.407 | 0.045 | O-Dealkylation |
| 0.205 | 0.191 | N-Glucuronidation |
| 0.120 | 0.181 | N-Dealkylation |
| 0.022 | 0.681 | N-Oxidation |
| 0.035 | 0.737 | Aliphatic Hydroxylation |
| 0.014 | 0.833 | Aromatic Hydroxylation |
| 0.037 | 0.969 | C-Oxidation |

Pa is the probability that this substance would undergo this reaction of biotransformation

Pi is the probability that this substance would not undergo this reaction of biotransformation.

The biotransformation reactions, that are experimentally observed, are highlighted

Table 2-2. Result of reacting atom prediction for codeine

| Atom number | Aliphatic  hydroxylation | Aromatic  hydroxylation | C-oxidation | N-oxidation | O-dealkylation | N-dealkylation | O-glucuronidation | N-glucuronidation |
| --- | --- | --- | --- | --- | --- | --- | --- | --- |
| 1 |  |  |  |  | 23(-0.953) | 23(-0.955) | 23(-0.934) |  |
| 2 |  |  |  |  | 19(-0.791) | 20(-0.893) | 19(-0.780) |  |
| 3 |  |  |  |  | 22(-0.910) | 18(-0.877) | 22(-0.887) |  |
| 4 |  |  |  |  | 10(-0.460) | 19(-0.888) | 13(-0.524) |  |
| 5 |  |  |  |  | 20(-0.867) | 22(-0.932) | 20(-0.786) |  |
| 6 |  |  |  |  | 4(-0.041) | 15(-0.788) | 4(-0.092) |  |
| 7 |  |  |  |  | 21(-0.879) | 14(-0.786) | 21(-0.862) |  |
| 8 |  |  |  |  | 15(-0.639) | 16(-0.805) | 16(-0.596) |  |
| 9 |  |  |  |  | 13(-0.559) | 10(-0.689) | 11(-0.445) |  |
| 10 |  |  |  |  | 14(-0.581) | 6(-0.530) | 15(-0.549) |  |
| 11 |  |  |  |  | 18(-0.774) | 1(0.783) | 18(-0.698) |  |
| 12 |  |  |  |  | 16(-0.649) | 9(-0.651) | 14(-0.545) |  |
| 13 |  |  |  |  | 11(-0.486) | 12(-0.723) | 9(-0.331) |  |
| 14 |  |  |  |  | 17(-0.755) | 21(-0.923) | 17(-0.625) |  |
| 15 |  |  |  |  | 9(-0.347) | 17(-0.844) | 12(-0.462) |  |
| 16 |  |  |  |  | 12(-0.516) | 5(-0.425) | 10(-0.400) |  |
| 17 |  |  |  |  | 8(-0.318) | 11(-0.705) | 8(-0.320) |  |
| 18 |  |  |  |  | 7(-0.271) | 13(-0.745) | 7(-0.304) |  |
| 19 |  |  |  |  | 3(-0.036) | 8(-0.606) | 1(0.584) |  |
| 20 |  |  |  |  | 1(0.608) | 7(-0.594) | 2(-0.040) |  |
| 21 |  |  |  |  | 6(-0.195) | 3(-0.143) | 6(-0.140) |  |
| 22 |  |  |  |  | 5(-0.076) | 4(-0.360) | 5(-0.114) |  |
| 23 |  |  |  |  | 2(-0.019) | 2(-0.049) | 3(-0.049) |  |
| 24 |  |  |  |  | 2(-0.019) | 2(-0.049) | 3(-0.049) |  |

The rank of the prediction is presented in the column.

DeltaP = Pa-Pi is presented in the brackets; Pa is the probability that this atom would be the reacting atom and Pi is the probability that it would not be the reacting atom of the specified reaction of biotransformation.

The real reacting atoms are highlighted.


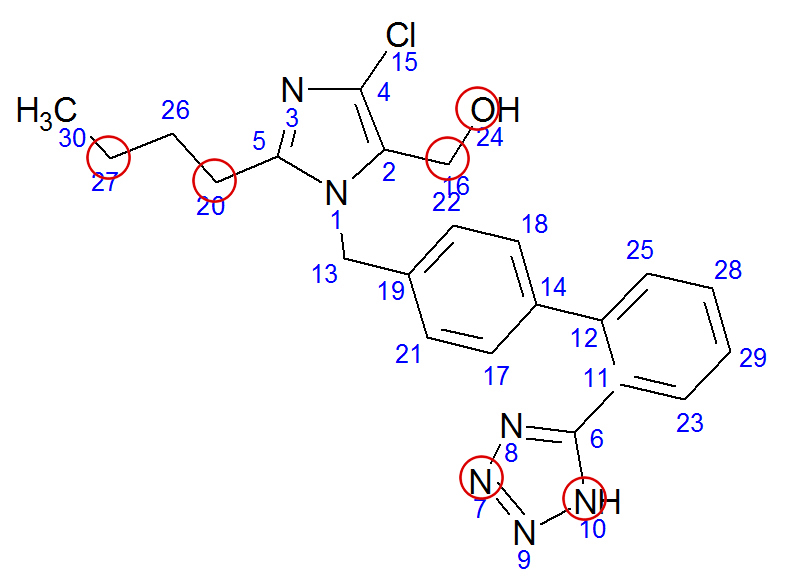


Figure 3. Structure of losartan

Table 3-1. Result of biotransformation class prediction for losartan

| Pa | Pi | Reaction |
| --- | --- | --- |
| 0.815 | 0.014 | C-Oxidation |
| 0.791 | 0.008 | N-Glucuronidation |
| 0.724 | 0.035 | Aliphatic Hydroxylation |
| 0.139 | 0.341 | N-Oxidation |
| 0.140 | 0.452 | Aromatic Hydroxylation |
| 0.058 | 0.387 | O-Glucuronidation |
| 0.074 | 0.428 | O-Dealkylation |
| 0.013 | 0.578 | N-Dealkylation |

Pa is the probability that this substance would undergo this reaction of biotransformation

Pi is the probability that this substance would not undergo this reaction of biotransformation.

The biotransformation reactions, that are experimentally observed, are highlighted

Table 3-2. Result of reacting atom prediction for losartan

| Atom number | Aliphatic  hydroxylation | Aromatic  hydroxylation | C-oxidation | N-oxidation | O-dealkylation | N-dealkylation | O-glucuronidation | N-glucuronidation |
| --- | --- | --- | --- | --- | --- | --- | --- | --- |
| 1 | 27(-0.918) |  | 24(-0.680) |  |  |  | 24(-0.550) | 4(-0.099) |
| 2 | 14(-0.744) |  | 20(-0.481) |  |  |  | 25(-0.558) | 27(-0.839) |
| 3 | 19(-0.775) |  | 13(-0.392) |  |  |  | 9(-0.315) | 6(-0.189) |
| 4 | 10(-0.653) |  | 9(-0.268) |  |  |  | 11(-0.348) | 24(-0.701) |
| 5 | 6(-0.427) |  | 18(-0.472) |  |  |  | 23(-0.541) | 26(-0.778) |
| 6 | 25(-0.907) |  | 27(-0.712) |  |  |  | 26(-0.639) | 8(-0.355) |
| 7 | 23(-0.876) |  | 17(-0.471) |  |  |  | 3(-0.205) | 3(-0.078) |
| 8 | 26(-0.908) |  | 22(-0.558) |  |  |  | 7(-0.275) | 2(-0.056) |
| 9 | 24(-0.906) |  | 21(-0.551) |  |  |  | 6(-0.261) | 5(-0.102) |
| 10 | 28(-0.953) |  | 25(-0.685) |  |  |  | 15(-0.434) | 1(0.591) |
| 11 | 22(-0.851) |  | 26(-0.698) |  |  |  | 27(-0.652) | 16(-0.610) |
| 12 | 21(-0.848) |  | 23(-0.562) |  |  |  | 18(-0.458) | 15(-0.592) |
| 13 | 5(-0.239) |  | 5(-0.065) |  |  |  | 12(-0.351) | 19(-0.681) |
| 14 | 15(-0.755) |  | 15(-0.449) |  |  |  | 13(-0.374) | 14(-0.581) |
| 15 | 8(-0.595) |  | 8(-0.154) |  |  |  | 2(-0.134) | 7(-0.295) |
| 16 | 7(-0.484) |  | 1(0.784) |  |  |  | 4(-0.211) | 25(-0.716) |
| 17 | 9(-0.614) |  | 15(-0.449) |  |  |  | 16(-0.446) | 17(-0.676) |
| 18 | 9(-0.614) |  | 15(-0.449) |  |  |  | 16(-0.446) | 17(-0.676) |
| 19 | 17(-0.764) |  | 12(-0.376) |  |  |  | 10(-0.335) | 13(-0.561) |
| 20 | 1(0.791) |  | 7(-0.128) |  |  |  | 20(-0.475) | 22(-0.692) |
| 21 | 16(-0.759) |  | 19(-0.476) |  |  |  | 17(-0.456) | 20(-0.687) |
| 22 | 16(-0.759) |  | 19(-0.476) |  |  |  | 17(-0.456) | 20(-0.687) |
| 23 | 13(-0.708) |  | 14(-0.426) |  |  |  | 21(-0.478) | 15(-0.592) |
| 24 | 18(-0.773) |  | 6(-0.116) |  |  |  | 1(0.506) | 11(-0.494) |
| 25 | 20(-0.800) |  | 16(-0.467) |  |  |  | 22(-0.502) | 23(-0.696) |
| 26 | 3(0.279) |  | 4(-0.025) |  |  |  | 19(-0.471) | 21(-0.691) |
| 27 | 2(0.387) |  | 2(0.010) |  |  |  | 14(-0.431) | 18(-0.677) |
| 28 | 12(-0.658) |  | 10(-0.357) |  |  |  | 8(-0.292) | 12(-0.525) |
| 29 | 11(-0.657) |  | 11(-0.360) |  |  |  | 8(-0.292) | 10(-0.482) |
| 30 | 4(0.227) |  | 3(0.004) |  |  |  | 5(-0.218) | 9(-0.440) |

The rank of the prediction is presented in the column.

DeltaP = Pa-Pi is presented in the brackets; Pa is the probability that this atom would be the reacting atom and Pi is the probability that it would not be the reacting atom of the specified reaction of biotransformation.

The real reacting atoms are highlighted.


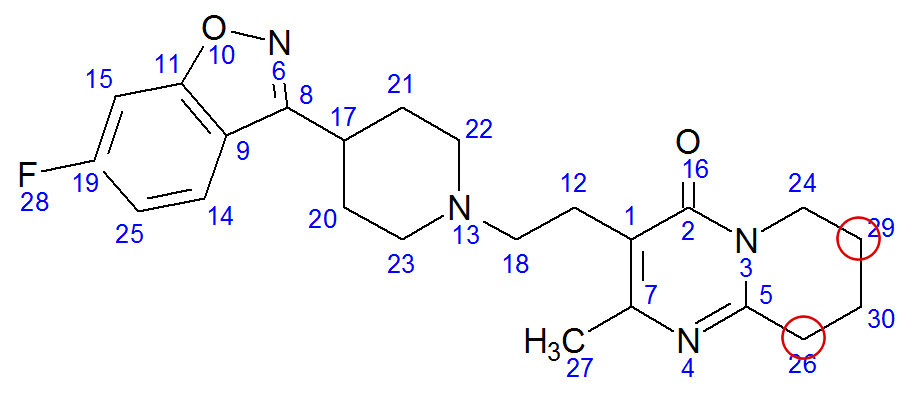


Figure4. Structure of risperidone

Table 4-1. Result of biotransformation class prediction for risperidone

| Pa | Pi | Reaction |
| --- | --- | --- |
| 0.826 | 0.018 | Aliphatic Hydroxylation |
| 0.209 | 0.142 | O-Dealkylation |
| 0.237 | 0.331 | C-Oxidation |
| 0.163 | 0.303 | N-Oxidation |
| 0.115 | 0.339 | N-Glucuronidation |
| 0.156 | 0.429 | Aromatic Hydroxylation |
| 0.047 | 0.343 | N-Dealkylation |
| 0.040 | 0.478 | O-Glucuronidation |

Pa is the probability that this substance would undergo this reaction of biotransformation

Pi is the probability that this substance would not undergo this reaction of biotransformation.

The biotransformation reactions, that are experimentally observed, are highlighted

Table 4-2. Result of reacting atom prediction for risperedone

| Atom number | Aliphatic  hydroxylation | Aromatic  hydroxylation | C-oxidation | N-oxidation | O-dealkylation | N-dealkylation | O-glucuronidation | N-glucuronidation |
| --- | --- | --- | --- | --- | --- | --- | --- | --- |
| 1 | 11(-0.384) |  |  |  |  |  |  |  |
| 2 | 19(-0.794) |  |  |  |  |  |  |  |
| 3 | 27(-0.920) |  |  |  |  |  |  |  |
| 4 | 16(-0.755) |  |  |  |  |  |  |  |
| 5 | 12(-0.476) |  |  |  |  |  |  |  |
| 6 | 23(-0.878) |  |  |  |  |  |  |  |
| 7 | 14(-0.633) |  |  |  |  |  |  |  |
| 8 | 18(-0.787) |  |  |  |  |  |  |  |
| 9 | 28(-0.926) |  |  |  |  |  |  |  |
| 10 | 24(-0.887) |  |  |  |  |  |  |  |
| 11 | 26(-0.900) |  |  |  |  |  |  |  |
| 12 | 3(0.131) |  |  |  |  |  |  |  |
| 13 | 20(-0.842) |  |  |  |  |  |  |  |
| 14 | 17(-0.775) |  |  |  |  |  |  |  |
| 15 | 22(-0.853) |  |  |  |  |  |  |  |
| 16 | 13(-0.632) |  |  |  |  |  |  |  |
| 17 | 10(-0.359) |  |  |  |  |  |  |  |
| 18 | 9(-0.217) |  |  |  |  |  |  |  |
| 19 | 25(-0.888) |  |  |  |  |  |  |  |
| 20 | 8(-0.135) |  |  |  |  |  |  |  |
| 21 | 8(-0.135) |  |  |  |  |  |  |  |
| 22 | 7(-0.100) |  |  |  |  |  |  |  |
| 23 | 7(-0.100) |  |  |  |  |  |  |  |
| 24 | 6(-0.054) |  |  |  |  |  |  |  |
| 25 | 21(-0.845) |  |  |  |  |  |  |  |
| 26 | 4(0.124) |  |  |  |  |  |  |  |
| 27 | 5(0.029) |  |  |  |  |  |  |  |
| 28 | 15(-0.729) |  |  |  |  |  |  |  |
| 29 | 1(0.441) |  |  |  |  |  |  |  |
| 30 | 2(0.162) |  |  |  |  |  |  |  |

The rank of the prediction is presented in the column.

DeltaP = Pa-Pi is presented in the brackets; Pa is the probability that this atom would be the reacting atom and Pi is the probability that it would not be the reacting atom of the specified reaction of biotransformation.

The real reacting atoms are highlighted.


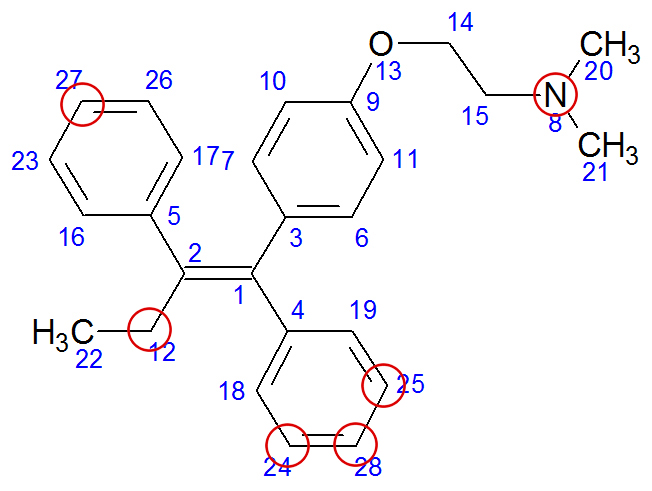


Figure 5. Structure of tamoxifen

Table 5-1. Result of biotransformation class prediction for tamoxifen

| Pa | Pi | Reaction |
| --- | --- | --- |
| 0.531 | 0.040 | N-Dealkylation |
| 0.414 | 0.204 | Aromatic Hydroxylation |
| 0.260 | 0.137 | N-Glucuronidation |
| 0.204 | 0.177 | O-Glucuronidation |
| 0.141 | 0.336 | N-Oxidation |
| 0.190 | 0.450 | C-Oxidation |
| 0.132 | 0.468 | Aliphatic Hydroxylation |
| 0.073 | 0.431 | O-Dealkylation |

Pa is the probability that this substance would undergo this reaction of biotransformation

Pi is the probability that this substance would not undergo this reaction of biotransformation.

The biotransformation reactions, that are experimentally observed, are highlighted.

Table 5-2. Result of reacting atom prediction for tamoxifen

| Atom number | Aliphatic  hydroxylation | Aromatic  hydroxylation | C-oxidation | N-oxidation | O-dealkylation | N-dealkylation | O-glucuronidation | N-glucuronidation |
| --- | --- | --- | --- | --- | --- | --- | --- | --- |
| 1 | 3(-0.032) | 3(-0.032) |  |  |  | 11(-0.817) |  | 6(-0.637) |
| 2 | 4(-0.147) | 4(-0.147) |  |  |  | 19(-0.947) |  | 18(-0.869) |
| 3 | 15(-0.916) | 15(-0.916) |  |  |  | 15(-0.857) |  | 13(-0.723) |
| 4 | 14(-0.904) | 14(-0.904) |  |  |  | 12(-0.829) |  | 10(-0.685) |
| 5 | 9(-0.771) | 9(-0.771) |  |  |  | 17(-0.866) |  | 14(-0.736) |
| 6 | 14(-0.904) | 14(-0.904) |  |  |  | 14(-0.855) |  | 15(-0.754) |
| 7 | 14(-0.904) | 14(-0.904) |  |  |  | 14(-0.855) |  | 15(-0.754) |
| 8 | 18(-0.991) | 18(-0.991) |  |  |  | 1(0.877) |  | 1(0.819) |
| 9 | 17(-0.976) | 17(-0.976) |  |  |  | 7(-0.742) |  | 7(-0.657) |
| 10 | 16(-0.958) | 16(-0.958) |  |  |  | 18(-0.885) |  | 17(-0.797) |
| 11 | 16(-0.958) | 16(-0.958) |  |  |  | 18(-0.885) |  | 17(-0.797) |
| 12 | 1(0.948) | 1(0.948) |  |  |  | 20(-0.965) |  | 19(-0.888) |
| 13 | 18(-0.991) | 18(-0.991) |  |  |  | 9(-0.776) |  | 8(-0.663) |
| 14 | 7(-0.483) | 7(-0.483) |  |  |  | 8(-0.751) |  | 11(-0.702) |
| 15 | 5(-0.262) | 5(-0.262) |  |  |  | 4(-0.621) |  | 5(-0.569) |
| 16 | 10(-0.829) | 10(-0.829) |  |  |  | 16(-0.863) |  | 16(-0.755) |
| 17 | 10(-0.829) | 10(-0.829) |  |  |  | 16(-0.863) |  | 16(-0.755) |
| 18 | 12(-0.877) | 12(-0.877) |  |  |  | 13(-0.830) |  | 12(-0.719) |
| 19 | 12(-0.877) | 12(-0.877) |  |  |  | 13(-0.830) |  | 12(-0.719) |
| 20 | 6(-0.409) | 6(-0.409) |  |  |  | 2(-0.320) |  | 2(-0.199) |
| 21 | 6(-0.409) | 6(-0.409) |  |  |  | 2(-0.320) |  | 2(-0.199) |
| 22 | 2(0.156) | 2(0.156) |  |  |  | 10(-0.811) |  | 9(-0.683) |
| 23 | 11(-0.849) | 11(-0.849) |  |  |  | 6(-0.731) |  | 5(-0.569) |
| 24 | 13(-0.891) | 13(-0.891) |  |  |  | 5(-0.680) |  | 4(-0.508) |
| 25 | 13(-0.891) | 13(-0.891) |  |  |  | 5(-0.680) |  | 4(-0.508) |
| 26 | 11(-0.849) | 11(-0.849) |  |  |  | 6(-0.731) |  | 5(-0.569) |
| 27 | 8(-0.758) | 8(-0.758) |  |  |  | 3(-0.522) |  | 3(-0.345) |
| 28 | 8(-0.758) | 8(-0.758) |  |  |  | 3(-0.522) |  | 3(-0.345) |

The rank of the prediction is presented in the column.

DeltaP = Pa-Pi is presented in the brackets; Pa is the probability that this atom would be the reacting atom and Pi is the probability that it would not be the reacting atom of the specified reaction of biotransformation.

The real reacting atoms are highlighted.


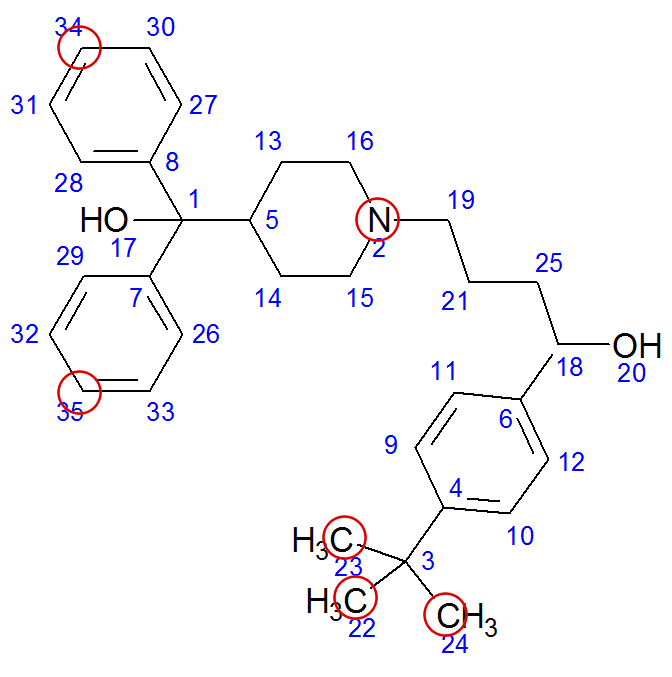


Figure 6. Structure of terfenadine.

Table 6-1. Result of biotransformation class prediction for terfenadine

| Pa | Pi | Reaction |
| --- | --- | --- |
| 0.915 | 0.004 | N-Dealkylation |
| 0.897 | 0.007 | C-Oxidation |
| 0.230 | 0.233 | N-Oxidation |
| 0.101 | 0.520 | Aliphatic Hydroxylation |
| 0.040 | 0.549 | N-Glucuronidation |
| 0.045 | 0.568 | O-Dealkylation |
| 0.020 | 0.666 | O-Glucuronidation |
| 0.028 | 0.747 | Aromatic Hydroxylation |

Pa is the probability that this substance would undergo this reaction of biotransformation

Pi is the probability that this substance would not undergo this reaction of biotransformation.

The biotransformation reactions, that are experimentally observed, are highlighted.

Table 6-2. Result of reacting atom prediction for terfenadine.

| Atom number | Aliphatic  hydroxylation | Aromatic  hydroxylation | C-oxidation | N-oxidation | O-dealkylation | N-dealkylation | O-glucuronidation | N-glucuronidation |
| --- | --- | --- | --- | --- | --- | --- | --- | --- |
| 1 | 9(0.003) | 12(-0.548) | 4(0.136) |  |  | 14(-0.435) |  |  |
| 2 | 19(-0.731) | 19(-0.859) | 21(-0.668) |  |  | 1(0.467) |  |  |
| 3 | 5(0.217) | 10(-0.301) | 3(0.142) |  |  | 5(-0.185) |  |  |
| 4 | 17(-0.344) | 5(-0.083) | 7(-0.009) |  |  | 7(-0.202) |  |  |
| 5 | 3(0.268) | 11(-0.470) | 17(-0.498) |  |  | 17(-0.537) |  |  |
| 6 | 20(-0.856) | 9(-0.297) | 18(-0.617) |  |  | 15(-0.478) |  |  |
| 7 | 13(-0.182) | 6(-0.151) | 20(-0.653) |  |  | 13(-0.422) |  |  |
| 8 | 13(-0.182) | 6(-0.151) | 20(-0.653) |  |  | 13(-0.422) |  |  |
| 9 | 16(-0.343) | 4(0.049) | 11(-0.214) |  |  | 12(-0.406) |  |  |
| 10 | 16(-0.343) | 4(0.049) | 11(-0.214) |  |  | 12(-0.406) |  |  |
| 11 | 18(-0.461) | 4(0.049) | 14(-0.346) |  |  | 13(-0.422) |  |  |
| 12 | 18(-0.461) | 4(0.049) | 14(-0.346) |  |  | 13(-0.422) |  |  |
| 13 | 4(0.230) | 14(-0.706) | 10(-0.171) |  |  | 16(-0.483) |  |  |
| 14 | 4(0.230) | 14(-0.706) | 10(-0.171) |  |  | 16(-0.483) |  |  |
| 15 | 6(0.175) | 17(-0.738) | 8(-0.099) |  |  | 8(-0.268) |  |  |
| 16 | 6(0.175) | 17(-0.738) | 8(-0.099) |  |  | 8(-0.268) |  |  |
| 17 | 12(-0.162) | 8(-0.253) | 16(-0.489) |  |  | 6(-0.189) |  |  |
| 18 | 10(-0.158) | 18(-0.850) | 2(0.159) |  |  | 18(-0.547) |  |  |
| 19 | 1(0.611) | 16(-0.715) | 5(0.067) |  |  | 5(-0.185) |  |  |
| 20 | 21(-0.896) | 15(-0.709) | 19(-0.650) |  |  | 9(-0.301) |  |  |
| 21 | 8(0.045) | 13(-0.656) | 6(0.022) |  |  | 11(-0.363) |  |  |
| 22 | 7(0.078) | 7(-0.187) | 1(0.247) |  |  | 3(-0.143) |  |  |
| 23 | 7(0.078) | 7(-0.187) | 1(0.247) |  |  | 3(-0.143) |  |  |
| 24 | 7(0.078) | 7(-0.187) | 1(0.247) |  |  | 3(-0.143) |  |  |
| 25 | 2(0.318) | 20(-0.881) | 12(-0.258) |  |  | 19(-0.629) |  |  |
| 26 | 14(-0.228) | 3(0.166) | 15(-0.358) |  |  | 10(-0.334) |  |  |
| 27 | 14(-0.228) | 3(0.166) | 15(-0.358) |  |  | 10(-0.334) |  |  |
| 28 | 14(-0.228) | 3(0.166) | 15(-0.358) |  |  | 10(-0.334) |  |  |
| 29 | 14(-0.228) | 3(0.166) | 15(-0.358) |  |  | 10(-0.334) |  |  |
| 30 | 15(-0.237) | 2(0.428) | 13(-0.297) |  |  | 4(-0.176) |  |  |
| 31 | 15(-0.237) | 2(0.428) | 13(-0.297) |  |  | 4(-0.176) |  |  |
| 32 | 15(-0.237) | 2(0.428) | 13(-0.297) |  |  | 4(-0.176) |  |  |
| 33 | 15(-0.237) | 2(0.428) | 13(-0.297) |  |  | 4(-0.176) |  |  |
| 34 | 11(-0.159) | 1(0.497) | 9(-0.153) |  |  | 2(-0.101) |  |  |
| 35 | 11(-0.159) | 1(0.497) | 9(-0.153) |  |  | 2(-0.101) |  |  |

The rank of the prediction is presented in the column.

DeltaP = Pa-Pi is presented in the brackets; Pa is the probability that this atom would be the reacting atom and Pi is the probability that it would not be the reacting atom of the specified reaction of biotransformation.

The real reacting atoms are highlighted.


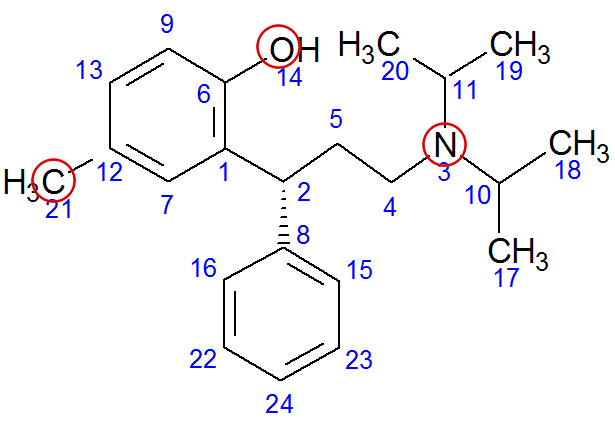


Figure 7. Structure of tolterodine

Table 7-1. Result of biotransformation class prediction for tolterodine

| Pa | Pi | Reaction |
| --- | --- | --- |
| 0.490 | 0.050 | C-Oxidation |
| 0.366 | 0.090 | O-Glucuronidation |
| 0.286 | 0.092 | N-Dealkylation |
| 0.229 | 0.294 | Aliphatic Hydroxylation |
| 0.171 | 0.408 | Aromatic Hydroxylation |
| 0.086 | 0.387 | O-Dealkylation |
| 0.043 | 0.544 | N-Glucuronidation |
| 0.025 | 0.655 | N-Oxidation |

Pa is the probability that this substance would undergo this reaction of biotransformation

Pi is the probability that this substance would not undergo this reaction of biotransformation.

The biotransformation reactions, that are experimentally observed, are highlighted.

Table 7-2. Result of reacting atom prediction for tolterodine

| Atom number | Aliphatic  hydroxylation | Aromatic  hydroxylation | C-oxidation | N-oxidation | O-dealkylation | N-dealkylation | O-glucuronidation | N-glucuronidation |
| --- | --- | --- | --- | --- | --- | --- | --- | --- |
| 1 | 18(-0.986) |  |  |  |  | 18(-0.986) | 18(-0.885) |  |
| 2 | 2(0.327) |  |  |  |  | 16(-0.931) | 17(-0.844) |  |
| 3 | 14(-0.904) |  |  |  |  | 1(0.866) | 14(-0.629) |  |
| 4 | 5(0.123) |  |  |  |  | 2(-0.266) | 15(-0.703) |  |
| 5 | 4(0.195) |  |  |  |  | 11(-0.842) | 16(-0.840) |  |
| 6 | 17(-0.976) |  |  |  |  | 17(-0.933) | 13(-0.582) |  |
| 7 | 10(-0.740) |  |  |  |  | 14(-0.877) | 10(-0.485) |  |
| 8 | 15(-0.948) |  |  |  |  | 10(-0.803) | 11(-0.503) |  |
| 9 | 13(-0.892) |  |  |  |  | 15(-0.887) | 7(-0.450) |  |
| 10 | 6(0.050) |  |  |  |  | 3(-0.324) | 6(-0.410) |  |
| 11 | 6(0.050) |  |  |  |  | 3(-0.324) | 6(-0.410) |  |
| 12 | 12(-0.852) |  |  |  |  | 13(-0.863) | 8(-0.459) |  |
| 13 | 8(-0.713) |  |  |  |  | 12(-0.853) | 12(-0.557) |  |
| 14 | 16(-0.966) |  |  |  |  | 8(-0.726) | 1(0.835) |  |
| 15 | 11(-0.750) |  |  |  |  | 9(-0.752) | 9(-0.474) |  |
| 16 | 11(-0.750) |  |  |  |  | 9(-0.752) | 9(-0.474) |  |
| 17 | 3(0.271) |  |  |  |  | 4(-0.328) | 3(-0.191) |  |
| 18 | 3(0.271) |  |  |  |  | 4(-0.328) | 3(-0.191) |  |
| 19 | 3(0.271) |  |  |  |  | 4(-0.328) | 3(-0.191) |  |
| 20 | 3(0.271) |  |  |  |  | 4(-0.328) | 3(-0.191) |  |
| 21 | 1(0.760) |  |  |  |  | 6(-0.580) | 4(-0.213) |  |
| 22 | 9(-0.724) |  |  |  |  | 7(-0.620) | 5(-0.282) |  |
| 23 | 9(-0.724) |  |  |  |  | 7(-0.620) | 5(-0.282) |  |
| 24 | 7(-0.480) |  |  |  |  | 5(-0.369) | 2(-0.156) |  |

The rank of the prediction is presented in the column.

DeltaP = Pa-Pi is presented in the brackets; Pa is the probability that this atom would be the reacting atom and Pi is the probability that it would not be the reacting atom of the specified reaction of biotransformation.

The real reacting atoms are highlighted.


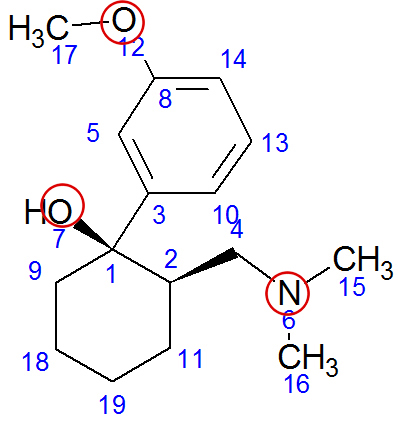


Figure 8. Structure of tramadol

Table 8-1. Result of biotransformation class prediction for tramadol

| Pa | Pi | Reaction |
| --- | --- | --- |
| 0.622 | 0.024 | N-Dealkylation |
| 0.521 | 0.028 | O-Dealkylation |
| 0.360 | 0.092 | O-Glucuronidation |
| 0.120 | 0.324 | N-Glucuronidation |
| 0.067 | 0.476 | N-Oxidation |
| 0.116 | 0.681 | C-Oxidation |
| 0.043 | 0.694 | Aromatic Hydroxylation |
| 0.020 | 0.829 | Aliphatic Hydroxylation |

Pa is the probability that this substance would undergo this reaction of biotransformation

Pi is the probability that this substance would not undergo this reaction of biotransformation.

The biotransformation reactions, that are experimentally observed, are highlighted.

Table 8-2. Result of reacting atom prediction for tramadol

| Atom number | Aliphatic  hydroxylation | Aromatic  hydroxylation | C-oxidation | N-oxidation | O-dealkylation | N-dealkylation | O-glucuronidation | N-glucuronidation |
| --- | --- | --- | --- | --- | --- | --- | --- | --- |
| 1 |  |  |  |  | 18(-0.984) | 18(-0.986) | 18(-0.958) |  |
| 2 |  |  |  |  | 17(-0.963) | 16(-0.937) | 17(-0.943) |  |
| 3 |  |  |  |  | 16(-0.918) | 17(-0.947) | 15(-0.734) |  |
| 4 |  |  |  |  | 12(-0.765) | 5(-0.580) | 16(-0.762) |  |
| 5 |  |  |  |  | 11(-0.753) | 15(-0.926) | 12(-0.536) |  |
| 6 |  |  |  |  | 4(-0.353) | 1(0.753) | 8(-0.365) |  |
| 7 |  |  |  |  | 6(-0.504) | 9(-0.778) | 1(0.651) |  |
| 8 |  |  |  |  | 7(-0.518) | 13(-0.886) | 9(-0.376) |  |
| 9 |  |  |  |  | 15(-0.890) | 12(-0.879) | 13(-0.591) |  |
| 10 |  |  |  |  | 13(-0.788) | 14(-0.899) | 11(-0.466) |  |
| 11 |  |  |  |  | 14(-0.796) | 10(-0.789) | 14(-0.709) |  |
| 12 |  |  |  |  | 1(0.840) | 8(-0.775) | 2(-0.033) |  |
| 13 |  |  |  |  | 5(-0.464) | 7(-0.741) | 5(-0.214) |  |
| 14 |  |  |  |  | 8(-0.569) | 11(-0.860) | 10(-0.406) |  |
| 15 |  |  |  |  | 3(-0.163) | 2(-0.164) | 4(-0.163) |  |
| 16 |  |  |  |  | 3(-0.163) | 2(-0.164) | 4(-0.163) |  |
| 17 |  |  |  |  | 2(-0.123) | 3(-0.478) | 3(-0.120) |  |
| 18 |  |  |  |  | 10(-0.582) | 6(-0.722) | 7(-0.243) |  |
| 19 |  |  |  |  | 9(-0.572) | 4(-0.557) | 6(-0.237) |  |

The rank of the prediction is presented in the column.

DeltaP = Pa-Pi is presented in the brackets; Pa is the probability that this atom would be the reacting atom and Pi is the probability that it would not be the reacting atom of the specified reaction of biotransformation.

The real reacting atoms are highlighted.


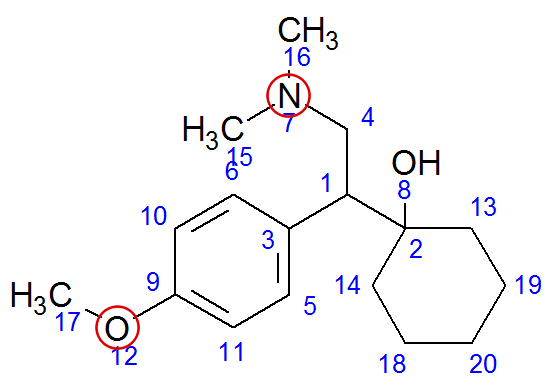


Figure 9. Structure of venlafaxine

Table 9-1. Result of biotransformation class prediction for venlafaxine

| Pa | Pi | Reaction |
| --- | --- | --- |
| 0.563 | 0.023 | O-Dealkylation |
| 0.558 | 0.035 | N-Dealkylation |
| 0.184 | 0.192 | O-Glucuronidation |
| 0.199 | 0.424 | C-Oxidation |
| 0.077 | 0.458 | N-Glucuronidation |
| 0.060 | 0.499 | N-Oxidation |
| 0.081 | 0.591 | Aromatic Hydroxylation |
| 0.062 | 0.626 | Aliphatic Hydroxylation |

Pa is the probability that this substance would undergo this reaction of biotransformation

Pi is the probability that this substance would not undergo this reaction of biotransformation.

The biotransformation reactions, that are experimentally observed, are highlighted.

Table 9-2. Result of reacting atom prediction for venlafaxine

| Atom number | Aliphatic  hydroxylation | Aromatic  hydroxylation | C-oxidation | N-oxidation | O-dealkylation | N-dealkylation | O-glucuronidation | N-glucuronidation |
| --- | --- | --- | --- | --- | --- | --- | --- | --- |
| 1 |  |  |  |  | 15(-0.979) | 15(-0.963) |  |  |
| 2 |  |  |  |  | 14(-0.933) | 14(-0.945) |  |  |
| 3 |  |  |  |  | 11(-0.809) | 13(-0.884) |  |  |
| 4 |  |  |  |  | 13(-0.903) | 8(-0.773) |  |  |
| 5 |  |  |  |  | 10(-0.735) | 10(-0.820) |  |  |
| 6 |  |  |  |  | 10(-0.735) | 10(-0.820) |  |  |
| 7 |  |  |  |  | 8(-0.534) | 1(0.818) |  |  |
| 8 |  |  |  |  | 3(-0.135) | 5(-0.719) |  |  |
| 9 |  |  |  |  | 5(-0.236) | 9(-0.794) |  |  |
| 10 |  |  |  |  | 9(-0.571) | 12(-0.874) |  |  |
| 11 |  |  |  |  | 9(-0.571) | 12(-0.874) |  |  |
| 12 |  |  |  |  | 1(0.826) | 7(-0.750) |  |  |
| 13 |  |  |  |  | 12(-0.835) | 11(-0.862) |  |  |
| 14 |  |  |  |  | 12(-0.835) | 11(-0.862) |  |  |
| 15 |  |  |  |  | 4(-0.213) | 2(-0.221) |  |  |
| 16 |  |  |  |  | 4(-0.213) | 2(-0.221) |  |  |
| 17 |  |  |  |  | 2(-0.113) | 4(-0.575) |  |  |
| 18 |  |  |  |  | 7(-0.493) | 6(-0.735) |  |  |
| 19 |  |  |  |  | 7(-0.493) | 6(-0.735) |  |  |
| 20 |  |  |  |  | 6(-0.257) | 3(-0.502) |  |  |

The rank of the prediction is presented in the column.

DeltaP = Pa-Pi is presented in the brackets; Pa is the probability that this atom would be the reacting atom and Pi is the probability that it would not be the reacting atom of the specified reaction of biotransformation.

The real reacting atoms are highlighted.


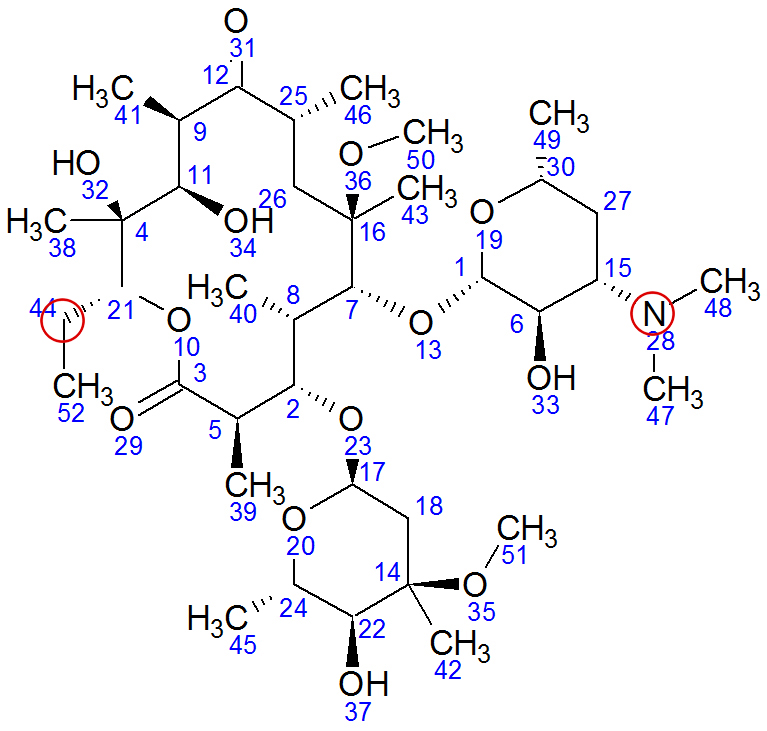


Figure 10. Structure of clarithromycin.

Table 10-1. Result of biotransformation class prediction for clarithromycin

| Pa | Pi | Reaction |
| --- | --- | --- |
| 0.835 | 0.006 | N-Dealkylation |
| 0.109 | 0.504 | Aliphatic Hydroxylation |
| 0.029 | 0.559 | O-Glucuronidation |
| 0.015 | 0.836 | O-Dealkylation |
| 0.010 | 0.864 | Aromatic Hydroxylation |
| 0.009 | 0.887 | N-Oxidation |
| 0.005 | 0.894 | N-Glucuronidation |
| 0.033 | 0.981 | C-Oxidation |

Pa is the probability that this substance would undergo this reaction of biotransformation

Pi is the probability that this substance would not undergo this reaction of biotransformation.

The biotransformation reactions, that are experimentally observed, are highlighted.

Table 10-2. Result of reacting atom prediction for clarithromycin.

| Atom number | Aliphatic  hydroxylation | Aromatic  hydroxylation | C-oxidation | N-oxidation | O-dealkylation | N-dealkylation | O-glucuronidation | N-glucuronidation |
| --- | --- | --- | --- | --- | --- | --- | --- | --- |
| 1 | 28(-0.435) |  |  |  |  | 36(-0.715) |  |  |
| 2 | 21(-0.260) |  |  |  |  | 35(-0.713) |  |  |
| 3 | 30(-0.490) |  |  |  |  | 34(-0.707) |  |  |
| 4 | 27(-0.410) |  |  |  |  | 44(-0.839) |  |  |
| 5 | 2(0.239) |  |  |  |  | 43(-0.831) |  |  |
| 6 | 40(-0.867) |  |  |  |  | 38(-0.720) |  |  |
| 7 | 19(-0.254) |  |  |  |  | 41(-0.790) |  |  |
| 8 | 9(0.016) |  |  |  |  | 35(-0.713) |  |  |
| 9 | 5(0.052) |  |  |  |  | 39(-0.775) |  |  |
| 10 | 36(-0.721) |  |  |  |  | 37(-0.716) |  |  |
| 11 | 26(-0.363) |  |  |  |  | 42(-0.803) |  |  |
| 12 | 29(-0.439) |  |  |  |  | 19(-0.563) |  |  |
| 13 | 38(-0.764) |  |  |  |  | 27(-0.637) |  |  |
| 14 | 17(-0.209) |  |  |  |  | 28(-0.649) |  |  |
| 15 | 23(-0.307) |  |  |  |  | 31(-0.683) |  |  |
| 16 | 22(-0.279) |  |  |  |  | 39(-0.775) |  |  |
| 17 | 18(-0.236) |  |  |  |  | 12(-0.488) |  |  |
| 18 | 15(-0.175) |  |  |  |  | 18(-0.558) |  |  |
| 19 | 41(-0.869) |  |  |  |  | 26(-0.630) |  |  |
| 20 | 38(-0.764) |  |  |  |  | 15(-0.508) |  |  |
| 21 | 12(-0.061) |  |  |  |  | 45(-0.880) |  |  |
| 22 | 20(-0.256) |  |  |  |  | 30(-0.670) |  |  |
| 23 | 35(-0.701) |  |  |  |  | 20(-0.572) |  |  |
| 24 | 16(-0.197) |  |  |  |  | 29(-0.654) |  |  |
| 25 | 4(0.065) |  |  |  |  | 32(-0.687) |  |  |
| 26 | 6(0.039) |  |  |  |  | 25(-0.618) |  |  |
| 27 | 8(0.031) |  |  |  |  | 24(-0.606) |  |  |
| 28 | 39(-0.771) |  |  |  |  | 1(0.450) |  |  |
| 29 | 32(-0.555) |  |  |  |  | 6(-0.422) |  |  |
| 30 | 24(-0.342) |  |  |  |  | 33(-0.700) |  |  |
| 31 | 31(-0.530) |  |  |  |  | 2(-0.245) |  |  |
| 32 | 34(-0.637) |  |  |  |  | 23(-0.597) |  |  |
| 33 | 38(-0.764) |  |  |  |  | 11(-0.462) |  |  |
| 34 | 37(-0.728) |  |  |  |  | 17(-0.517) |  |  |
| 35 | 33(-0.627) |  |  |  |  | 5(-0.385) |  |  |
| 36 | 33(-0.627) |  |  |  |  | 17(-0.517) |  |  |
| 37 | 33(-0.627) |  |  |  |  | 5(-0.385) |  |  |
| 38 | 13(-0.080) |  |  |  |  | 22(-0.591) |  |  |
| 39 | 7(0.032) |  |  |  |  | 14(-0.507) |  |  |
| 40 | 7(0.032) |  |  |  |  | 16(-0.509) |  |  |
| 41 | 10(-0.028) |  |  |  |  | 9(-0.443) |  |  |
| 42 | 11(-0.032) |  |  |  |  | 3(-0.318) |  |  |
| 43 | 11(-0.032) |  |  |  |  | 10(-0.446) |  |  |
| 44 | 1(0.283) |  |  |  |  | 40(-0.786) |  |  |
| 45 | 6(0.039) |  |  |  |  | 7(-0.440) |  |  |
| 46 | 7(0.032) |  |  |  |  | 8(-0.442) |  |  |
| 47 | 14(-0.132) |  |  |  |  | 4(-0.344) |  |  |
| 48 | 14(-0.132) |  |  |  |  | 4(-0.344) |  |  |
| 49 | 3(0.104) |  |  |  |  | 13(-0.503) |  |  |
| 50 | 25(-0.344) |  |  |  |  | 3(-0.318) |  |  |
| 51 | 25(-0.344) |  |  |  |  | 3(-0.318) |  |  |
| 52 | 3(0.104) |  |  |  |  | 21(-0.586) |  |  |

The rank of the prediction is presented in the column.

DeltaP = Pa-Pi is presented in the brackets; Pa is the probability that this atom would be the reacting atom and Pi is the probability that it would not be the reacting atom of the specified reaction of biotransformation.

The real reacting atoms are highlighted.


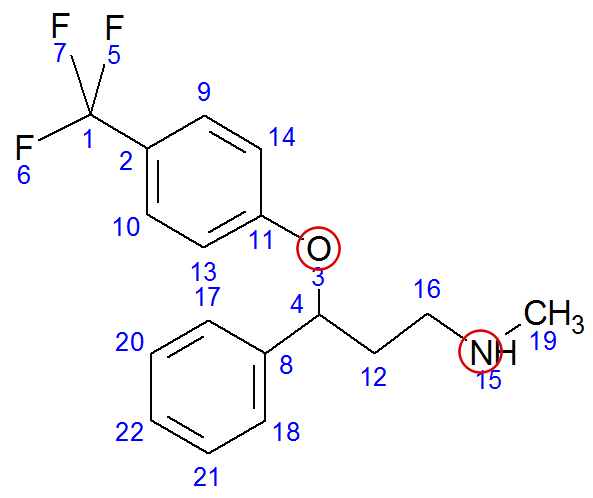


Figure 11. Structure of fluoxetine

Table 11-1. Result of biotransformation class prediction for fluoxetine

| Pa | Pi | Reaction |
| --- | --- | --- |
| 0.416 | 0.058 | N-Dealkylation |
| 0.434 | 0.185 | Aromatic Hydroxylation |
| 0.272 | 0.258 | C-Oxidation |
| 0.152 | 0.223 | O-Glucuronidation |
| 0.115 | 0.295 | O-Dealkylation |
| 0.116 | 0.335 | N-Glucuronidation |
| 0.093 | 0.415 | N-Oxidation |
| 0.029 | 0.767 | Aliphatic Hydroxylation |

Pa is the probability that this substance would undergo this reaction of biotransformation

Pi is the probability that this substance would not undergo this reaction of biotransformation.

The biotransformation reactions, that are experimentally observed, are highlighted.

Table 11-2. Result of reacting atom prediction for fluoxetine

| Atom number | Aliphatic  hydroxylation | Aromatic  hydroxylation | C-oxidation | N-oxidation | O-dealkylation | N-dealkylation | O-glucuronidation | N-glucuronidation |
| --- | --- | --- | --- | --- | --- | --- | --- | --- |
| 1 |  |  |  |  | 5(-0.363) | 5(-0.674) |  |  |
| 2 |  |  |  |  | 7(-0.563) | 8(-0.812) |  |  |
| 3 |  |  |  |  | 1(0.337) | 15(-0.956) |  |  |
| 4 |  |  |  |  | 13(-0.774) | 16(-0.977) |  |  |
| 5 |  |  |  |  | 2(-0.187) | 3(-0.440) |  |  |
| 6 |  |  |  |  | 2(-0.187) | 3(-0.440) |  |  |
| 7 |  |  |  |  | 2(-0.187) | 3(-0.440) |  |  |
| 8 |  |  |  |  | 6(-0.403) | 12(-0.917) |  |  |
| 9 |  |  |  |  | 12(-0.709) | 9(-0.878) |  |  |
| 10 |  |  |  |  | 12(-0.709) | 9(-0.878) |  |  |
| 11 |  |  |  |  | 3(-0.196) | 11(-0.906) |  |  |
| 12 |  |  |  |  | 14(-0.837) | 14(-0.951) |  |  |
| 13 |  |  |  |  | 9(-0.601) | 13(-0.929) |  |  |
| 14 |  |  |  |  | 9(-0.601) | 13(-0.929) |  |  |
| 15 |  |  |  |  | 16(-0.879) | 1(0.892) |  |  |
| 16 |  |  |  |  | 15(-0.854) | 6(-0.754) |  |  |
| 17 |  |  |  |  | 10(-0.630) | 10(-0.891) |  |  |
| 18 |  |  |  |  | 10(-0.630) | 10(-0.891) |  |  |
| 19 |  |  |  |  | 11(-0.652) | 2(-0.252) |  |  |
| 20 |  |  |  |  | 8(-0.581) | 7(-0.800) |  |  |
| 21 |  |  |  |  | 8(-0.581) | 7(-0.800) |  |  |
| 22 |  |  |  |  | 4(-0.337) | 4(-0.628) |  |  |

The rank of the prediction is presented in the column.

DeltaP = Pa-Pi is presented in the brackets; Pa is the probability that this atom would be the reacting atom and Pi is the probability that it would not be the reacting atom of the specified reaction of biotransformation.

The real reacting atoms are highlighted.


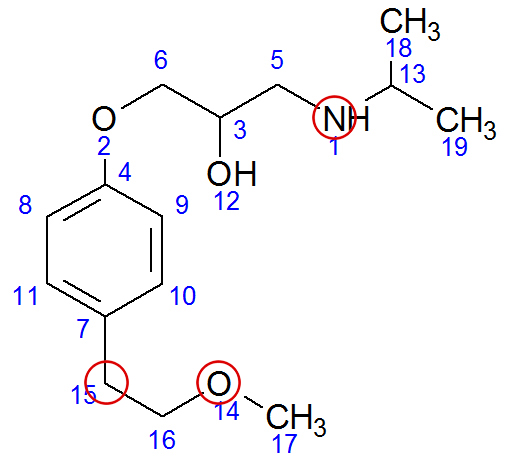


Figure 12. Structure of metoprolol

Table 12-1. Result of biotransformation class prediction for metoprolol

| Pa | Pi | Reaction |
| --- | --- | --- |
| 0.441 | 0.038 | O-Dealkylation |
| 0.398 | 0.219 | Aromatic Hydroxylation |
| 0.284 | 0.129 | O-Glucuronidation |
| 0.293 | 0.223 | C-Oxidation |
| 0.120 | 0.182 | N-Dealkylation |
| 0.166 | 0.395 | Aliphatic Hydroxylation |
| 0.072 | 0.459 | N-Oxidation |
| 0.023 | 0.613 | N-Glucuronidation |

Pa is the probability that this substance would undergo this reaction of biotransformation

Pi is the probability that this substance would not undergo this reaction of biotransformation.

The biotransformation reactions, that are experimentally observed, are highlighted.

Table 12-2. Result of reacting atom prediction for metoprolol

| Atom number | Aliphatic  hydroxylation | Aromatic  hydroxylation | C-oxidation | N-oxidation | O-dealkylation | N-dealkylation | O-glucuronidation | N-glucuronidation |
| --- | --- | --- | --- | --- | --- | --- | --- | --- |
| 1 | 16(-0.978) |  |  |  | 16(-0.913) | 1(0.805) |  |  |
| 2 | 14(-0.902) |  |  |  | 2(0.343) | 8(-0.661) |  |  |
| 3 | 9(-0.545) |  |  |  | 14(-0.898) | 13(-0.809) |  |  |
| 4 | 13(-0.790) |  |  |  | 5(-0.378) | 9(-0.757) |  |  |
| 5 | 6(-0.291) |  |  |  | 15(-0.905) | 7(-0.657) |  |  |
| 6 | 7(-0.361) |  |  |  | 12(-0.706) | 10(-0.790) |  |  |
| 7 | 8(-0.469) |  |  |  | 7(-0.561) | 12(-0.803) |  |  |
| 8 | 12(-0.735) |  |  |  | 9(-0.617) | 15(-0.847) |  |  |
| 9 | 12(-0.735) |  |  |  | 9(-0.617) | 15(-0.847) |  |  |
| 10 | 10(-0.599) |  |  |  | 11(-0.678) | 16(-0.859) |  |  |
| 11 | 10(-0.599) |  |  |  | 11(-0.678) | 16(-0.859) |  |  |
| 12 | 15(-0.917) |  |  |  | 4(-0.176) | 6(-0.634) |  |  |
| 13 | 3(0.079) |  |  |  | 13(-0.861) | 4(-0.475) |  |  |
| 14 | 11(-0.617) |  |  |  | 1(0.673) | 5(-0.625) |  |  |
| 15 | 1(0.737) |  |  |  | 10(-0.620) | 14(-0.834) |  |  |
| 16 | 4(-0.038) |  |  |  | 6(-0.545) | 11(-0.792) |  |  |
| 17 | 5(-0.283) |  |  |  | 3(-0.168) | 3(-0.384) |  |  |
| 18 | 2(0.174) |  |  |  | 8(-0.607) | 2(-0.308) |  |  |
| 19 | 2(0.174) |  |  |  | 8(-0.607) | 2(-0.308) |  |  |

The rank of the prediction is presented in the column.

DeltaP = Pa-Pi is presented in the brackets; Pa is the probability that this atom would be the reacting atom and Pi is the probability that it would not be the reacting atom of the specified reaction of biotransformation.

The real reacting atoms are highlighted.


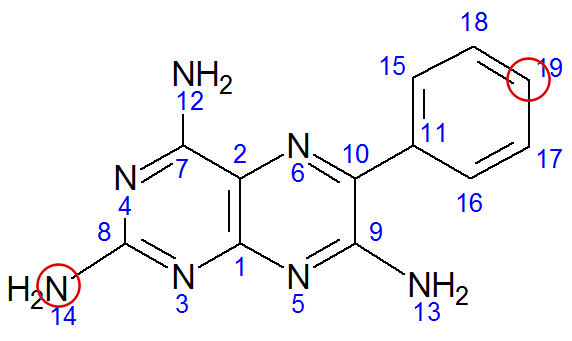


Figure 13. Structure of triamterene

Table 13-1. Result of biotransformation class prediction for triamterene

| Pa | Pi | Reaction |
| --- | --- | --- |
| 0.859 | 0.005 | N-Glucuronidation |
| 0.658 | 0.026 | N-Oxidation |
| 0.608 | 0.077 | Aromatic Hydroxylation |
| 0.246 | 0.312 | C-Oxidation |
| 0.147 | 0.438 | Aliphatic Hydroxylation |
| 0.007 | 0.725 | N-Dealkylation |

Pa is the probability that this substance would undergo this reaction of biotransformation

Pi is the probability that this substance would not undergo this reaction of biotransformation.

The biotransformation reactions, that are experimentally observed, are highlighted.

Table 13-2. Result of reacting atom prediction for triamterene

| Atom number | Aliphatic  hydroxylation | Aromatic  hydroxylation | C-oxidation | N-oxidation | O-dealkylation | N-dealkylation | O-glucuronidation | N-glucuronidation |
| --- | --- | --- | --- | --- | --- | --- | --- | --- |
| 1 |  | 12(-0.868) |  |  |  |  |  | 11(-0.902) |
| 2 |  | 8(-0.619) |  |  |  |  |  | 14(-0.947) |
| 3 |  | 17(-0.964) |  |  |  |  |  | 4(0.301) |
| 4 |  | 16(-0.962) |  |  |  |  |  | 5(0.153) |
| 5 |  | 10(-0.760) |  |  |  |  |  | 6(0.004) |
| 6 |  | 14(-0.899) |  |  |  |  |  | 7(-0.240) |
| 7 |  | 11(-0.850) |  |  |  |  |  | 12(-0.917) |
| 8 |  | 15(-0.954) |  |  |  |  |  | 8(-0.748) |
| 9 |  | 6(-0.467) |  |  |  |  |  | 16(-0.967) |
| 10 |  | 5(-0.334) |  |  |  |  |  | 17(-0.978) |
| 11 |  | 4(-0.175) |  |  |  |  |  | 13(-0.943) |
| 12 |  | 9(-0.739) |  |  |  |  |  | 2(0.476) |
| 13 |  | 7(-0.492) |  |  |  |  |  | 3(0.392) |
| 14 |  | 13(-0.892) |  |  |  |  |  | 1(0.839) |
| 15 |  | 3(0.381) |  |  |  |  |  | 15(-0.959) |
| 16 |  | 3(0.381) |  |  |  |  |  | 15(-0.959) |
| 17 |  | 1(0.810) |  |  |  |  |  | 10(-0.879) |
| 18 |  | 1(0.810) |  |  |  |  |  | 10(-0.879) |
| 19 |  | 2(0.747) |  |  |  |  |  | 9(-0.805) |

The rank of the prediction is presented in the column.

DeltaP = Pa-Pi is presented in the brackets; Pa is the probability that this atom would be the reacting atom and Pi is the probability that it would not be the reacting atom of the specified reaction of biotransformation.

The real reacting atoms are highlighted.


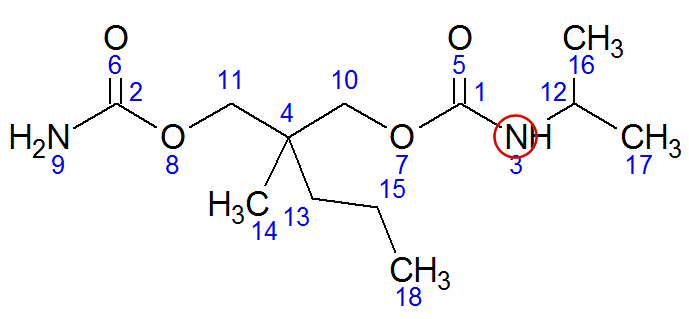


Figure 14. Structure of carisoprodol.

Table 14-1. Result of biotransformation class prediction for carisoprodol

| Pa | Pi | Reaction |
| --- | --- | --- |
| 0.723 | 0.035 | Aliphatic Hydroxylation |
| 0.281 | 0.189 | N-Oxidation |
| 0.293 | 0.304 | Aromatic Hydroxylation |
| 0.086 | 0.230 | N-Dealkylation |
| 0.117 | 0.289 | O-Dealkylation |
| 0.072 | 0.344 | O-Glucuronidation |
| 0.156 | 0.554 | C-Oxidation |
| 0.065 | 0.494 | N-Glucuronidation |

Pa is the probability that this substance would undergo this reaction of biotransformation

Pi is the probability that this substance would not undergo this reaction of biotransformation.

The biotransformation reactions, that are experimentally observed, are highlighted.

Table 14-2. Result of reacting atom prediction for carisoprodol.

| Atom number | Aliphatic  hydroxylation | Aromatic  hydroxylation | C-oxidation | N-oxidation | O-dealkylation | N-dealkylation | O-glucuronidation | N-glucuronidation |
| --- | --- | --- | --- | --- | --- | --- | --- | --- |
| 1 |  |  |  |  |  | 9(-0.804) |  |  |
| 2 |  |  |  |  |  | 13(-0.895) |  |  |
| 3 |  |  |  |  |  | 1(0.871) |  |  |
| 4 |  |  |  |  |  | 15(-0.957) |  |  |
| 5 |  |  |  |  |  | 3(-0.537) |  |  |
| 6 |  |  |  |  |  | 4(-0.649) |  |  |
| 7 |  |  |  |  |  | 6(-0.725) |  |  |
| 8 |  |  |  |  |  | 8(-0.800) |  |  |
| 9 |  |  |  |  |  | 2(0.047) |  |  |
| 10 |  |  |  |  |  | 12(-0.845) |  |  |
| 11 |  |  |  |  |  | 12(-0.845) |  |  |
| 12 |  |  |  |  |  | 11(-0.829) |  |  |
| 13 |  |  |  |  |  | 16(-0.958) |  |  |
| 14 |  |  |  |  |  | 5(-0.721) |  |  |
| 15 |  |  |  |  |  | 14(-0.932) |  |  |
| 16 |  |  |  |  |  | 7(-0.752) |  |  |
| 17 |  |  |  |  |  | 7(-0.752) |  |  |
| 18 |  |  |  |  |  | 10(-0.814) |  |  |

The rank of the prediction is presented in the column.

DeltaP = Pa-Pi is presented in the brackets; Pa is the probability that this atom would be the reacting atom and Pi is the probability that it would not be the reacting atom of the specified reaction of biotransformation.

The real reacting atoms are highlighted.


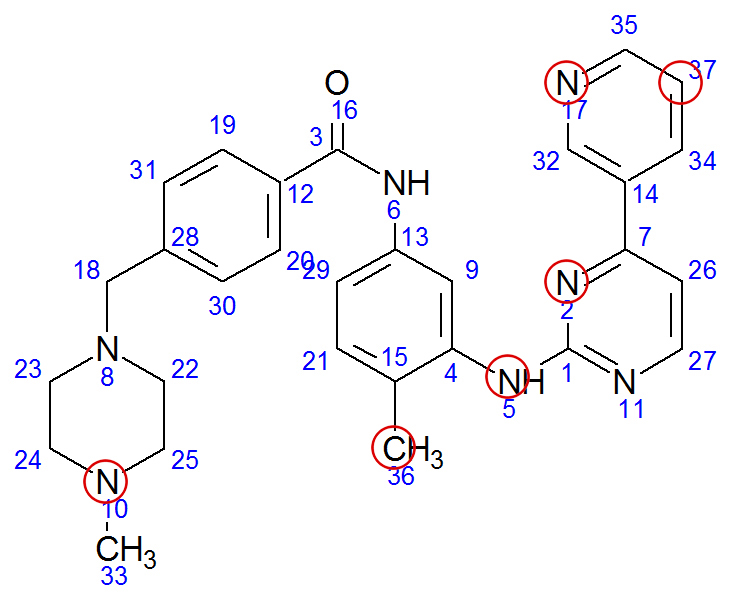


Figure 15. Structure of imatinib.

Table 15-1. Result of biotransformation class prediction for imatinib

| Pa | Pi | Reaction |
| --- | --- | --- |
| 0.833 | 0.005 | N-Glucuronidation |
| 0.749 | 0.014 | N-Oxidation |
| 0.530 | 0.117 | Aromatic Hydroxylation |
| 0.448 | 0.143 | Aliphatic Hydroxylation |
| 0.396 | 0.094 | C-Oxidation |
| 0.301 | 0.087 | N-Dealkylation |
| 0.084 | 0.395 | O-Dealkylation |
| 0.007 | 0.847 | O-Glucuronidation |

Pa is the probability that this substance would undergo this reaction of biotransformation

Pi is the probability that this substance would not undergo this reaction of biotransformation.

The biotransformation reactions, that are experimentally observed, are highlighted

Table 15-2. Result of reacting atom prediction for imatininb.

| Atom number | Aliphatic  hydroxylation | Aromatic  hydroxylation | C-oxidation | N-oxidation | O-dealkylation | N-dealkylation | O-glucuronidation | N-glucuronidation |
| --- | --- | --- | --- | --- | --- | --- | --- | --- |
| 1 | 28(-0.593) | 28(-0.567) |  | 22(-0.773) |  | 26(-0.582) |  | 14(-0.535) |
| 2 | 29(-0.606) | 23(-0.476) |  | 7(-0.060) |  | 7(-0.146) |  | 7(-0.073) |
| 3 | 6(-0.165) | 31(-0.631) |  | 31(-0.862) |  | 28(-0.666) |  | 26(-0.660) |
| 4 | 26(-0.460) | 22(-0.419) |  | 28(-0.815) |  | 30(-0.682) |  | 32(-0.754) |
| 5 | 31(-0.758) | 30(-0.586) |  | 4(0.121) |  | 4(-0.019) |  | 2(0.160) |
| 6 | 29(-0.606) | 32(-0.682) |  | 6(-0.057) |  | 3(0.021) |  | 4(0.020) |
| 7 | 21(-0.332) | 15(-0.257) |  | 26(-0.786) |  | 29(-0.677) |  | 28(-0.665) |
| 8 | 22(-0.354) | 24(-0.477) |  | 3(0.361) |  | 2(0.186) |  | 6(-0.049) |
| 9 | 18(-0.281) | 10(-0.127) |  | 11(-0.674) |  | 15(-0.459) |  | 21(-0.600) |
| 10 | 25(-0.412) | 26(-0.553) |  | 1(0.385) |  | 1(0.267) |  | 3(0.136) |
| 11 | 27(-0.526) | 25(-0.518) |  | 5(-0.011) |  | 8(-0.181) |  | 5(0.002) |
| 12 | 13(-0.266) | 14(-0.241) |  | 27(-0.809) |  | 19(-0.544) |  | 12(-0.518) |
| 13 | 23(-0.405) | 13(-0.222) |  | 21(-0.766) |  | 17(-0.504) |  | 29(-0.674) |
| 14 | 30(-0.678) | 12(-0.202) |  | 25(-0.785) |  | 31(-0.744) |  | 30(-0.711) |
| 15 | 20(-0.327) | 18(-0.324) |  | 24(-0.783) |  | 27(-0.636) |  | 31(-0.745) |
| 16 | 8(-0.220) | 19(-0.335) |  | 10(-0.669) |  | 9(-0.276) |  | 9(-0.298) |
| 17 | 24(-0.411) | 20(-0.344) |  | 2(0.381) |  | 5(-0.125) |  | 1(0.211) |
| 18 | 4(0.123) | 21(-0.365) |  | 12(-0.697) |  | 11(-0.320) |  | 11(-0.509) |
| 19 | 14(-0.267) | 8(-0.074) |  | 30(-0.825) |  | 25(-0.580) |  | 23(-0.623) |
| 20 | 14(-0.267) | 8(-0.074) |  | 30(-0.825) |  | 25(-0.580) |  | 23(-0.623) |
| 21 | 11(-0.257) | 9(-0.075) |  | 16(-0.736) |  | 23(-0.573) |  | 27(-0.664) |
| 22 | 2(0.223) | 27(-0.559) |  | 18(-0.744) |  | 13(-0.436) |  | 20(-0.589) |
| 23 | 2(0.223) | 27(-0.559) |  | 18(-0.744) |  | 13(-0.436) |  | 20(-0.589) |
| 24 | 5(0.066) | 29(-0.580) |  | 17(-0.743) |  | 14(-0.442) |  | 19(-0.583) |
| 25 | 5(0.066) | 29(-0.580) |  | 17(-0.743) |  | 14(-0.442) |  | 19(-0.583) |
| 26 | 10(-0.252) | 4(0.160) |  | 13(-0.721) |  | 22(-0.570) |  | 17(-0.565) |
| 27 | 9(-0.223) | 5(0.025) |  | 20(-0.760) |  | 26(-0.582) |  | 15(-0.545) |
| 28 | 12(-0.259) | 13(-0.222) |  | 12(-0.697) |  | 12(-0.327) |  | 11(-0.509) |
| 29 | 12(-0.259) | 2(0.347) |  | 15(-0.735) |  | 24(-0.574) |  | 25(-0.645) |
| 30 | 15(-0.269) | 7(-0.071) |  | 29(-0.816) |  | 18(-0.543) |  | 24(-0.626) |
| 31 | 15(-0.269) | 7(-0.071) |  | 29(-0.816) |  | 18(-0.543) |  | 24(-0.626) |
| 32 | 7(-0.215) | 11(-0.136) |  | 14(-0.722) |  | 16(-0.501) |  | 13(-0.534) |
| 33 | 3(0.135) | 17(-0.314) |  | 8(-0.512) |  | 6(-0.145) |  | 8(-0.287) |
| 34 | 17(-0.279) | 6(0.000) |  | 23(-0.781) |  | 23(-0.573) |  | 22(-0.610) |
| 35 | 16(-0.275) | 3(0.186) |  | 19(-0.751) |  | 21(-0.557) |  | 16(-0.553) |
| 36 | 1(0.474) | 16(-0.311) |  | 9(-0.560) |  | 10(-0.316) |  | 10(-0.432) |
| 37 | 19(-0.286) | 1(0.425) |  | 17(-0.743) |  | 20(-0.547) |  | 18(-0.573) |

The rank of the prediction is presented in the column.

DeltaP = Pa-Pi is presented in the brackets; Pa is the probability that this atom would be the reacting atom and Pi is the probability that it would not be the reacting atom of the specified reaction of biotransformation.

The real reacting atoms are highlighted.


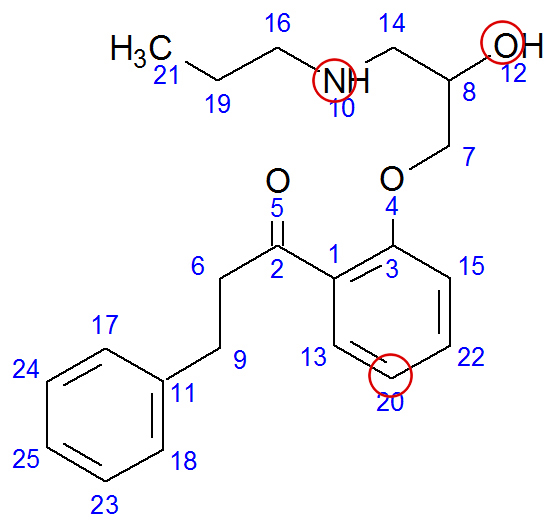


Figure 16. Structure of propafenone.

Table 16-1. Result of biotransformation class prediction for propafenone

| Pa | Pi | Reaction |
| --- | --- | --- |
| 0.454 | 0.166 | Aromatic Hydroxylation |
| 0.355 | 0.093 | O-Glucuronidation |
| 0.321 | 0.180 | C-Oxidation |
| 0.185 | 0.129 | N-Dealkylation |
| 0.161 | 0.204 | O-Dealkylation |
| 0.150 | 0.431 | Aliphatic Hydroxylation |
| 0.056 | 0.513 | N-Oxidation |
| 0.025 | 0.598 | N-Glucuronidation |

Pa is the probability that this substance would undergo this reaction of biotransformation

Pi is the probability that this substance would not undergo this reaction of biotransformation.

The biotransformation reactions, that are experimentally observed, are highlighted

Table 16-2. Result of reacting atom prediction for propafenone.

| Atom number | Aliphatic  hydroxylation | Aromatic  hydroxylation | C-oxidation | N-oxidation | O-dealkylation | N-dealkylation | O-glucuronidation | N-glucuronidation |
| --- | --- | --- | --- | --- | --- | --- | --- | --- |
| 1 |  | 8(-0.290) |  |  |  | 23(-0.857) | 23(-0.709) |  |
| 2 |  | 16(-0.677) |  |  |  | 20(-0.768) | 21(-0.646) |  |
| 3 |  | 12(-0.426) |  |  |  | 22(-0.841) | 20(-0.636) |  |
| 4 |  | 17(-0.731) |  |  |  | 15(-0.676) | 2(-0.033) |  |
| 5 |  | 10(-0.311) |  |  |  | 5(-0.307) | 3(-0.090) |  |
| 6 |  | 14(-0.484) |  |  |  | 21(-0.795) | 22(-0.649) |  |
| 7 |  | 20(-0.825) |  |  |  | 18(-0.747) | 14(-0.441) |  |
| 8 |  | 23(-0.939) |  |  |  | 19(-0.749) | 19(-0.589) |  |
| 9 |  | 13(-0.441) |  |  |  | 14(-0.659) | 13(-0.432) |  |
| 10 |  | 21(-0.831) |  |  |  | 1(0.693) | 10(-0.344) |  |
| 11 |  | 9(-0.293) |  |  |  | 12(-0.585) | 9(-0.336) |  |
| 12 |  | 18(-0.734) |  |  |  | 7(-0.433) | 1(0.675) |  |
| 13 |  | 6(-0.004) |  |  |  | 16(-0.689) | 16(-0.488) |  |
| 14 |  | 22(-0.889) |  |  |  | 9(-0.505) | 18(-0.504) |  |
| 15 |  | 5(0.241) |  |  |  | 17(-0.709) | 15(-0.478) |  |
| 16 |  | 19(-0.740) |  |  |  | 3(-0.270) | 17(-0.498) |  |
| 17 |  | 7(-0.006) |  |  |  | 13(-0.649) | 12(-0.409) |  |
| 18 |  | 7(-0.006) |  |  |  | 13(-0.649) | 12(-0.409) |  |
| 19 |  | 15(-0.617) |  |  |  | 6(-0.388) | 11(-0.394) |  |
| 20 |  | 3(0.312) |  |  |  | 10(-0.509) | 8(-0.262) |  |
| 21 |  | 11(-0.367) |  |  |  | 2(-0.200) | 5(-0.183) |  |
| 22 |  | 1(0.471) |  |  |  | 11(-0.522) | 7(-0.243) |  |
| 23 |  | 4(0.276) |  |  |  | 8(-0.494) | 6(-0.242) |  |
| 24 |  | 4(0.276) |  |  |  | 8(-0.494) | 6(-0.242) |  |
| 25 |  | 2(0.334) |  |  |  | 4(-0.287) | 4(-0.147) |  |

The rank of the prediction is presented in the column.

DeltaP = Pa-Pi is presented in the brackets; Pa is the probability that this atom would be the reacting atom and Pi is the probability that it would not be the reacting atom of the specified reaction of biotransformation.

The real reacting atoms are highlighted.


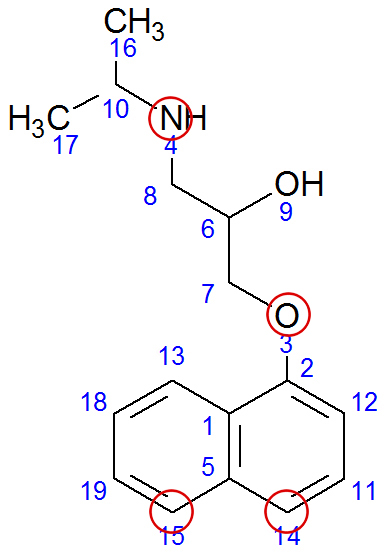


Figure 17. Structure of propranolol.

Table 17-1. Result of biotransformation class prediction for propranolol

| Pa | Pi | Reaction |
| --- | --- | --- |
| 0.454 | 0.166 | Aromatic Hydroxylation |
| 0.355 | 0.093 | O-Glucuronidation |
| 0.321 | 0.180 | C-Oxidation |
| 0.185 | 0.129 | N-Dealkylation |
| 0.161 | 0.204 | O-Dealkylation |
| 0.150 | 0.431 | Aliphatic Hydroxylation |
| 0.056 | 0.513 | N-Oxidation |
| 0.025 | 0.598 | N-Glucuronidation |

Pa is the probability that this substance would undergo this reaction of biotransformation

Pi is the probability that this substance would not undergo this reaction of biotransformation.

The biotransformation reactions, that are experimentally observed, are highlighted

Table 17-2. Result of reacting atom prediction for propranolol.

| Atom number | Aliphatic  hydroxylation | Aromatic  hydroxylation | C-oxidation | N-oxidation | O-dealkylation | N-dealkylation | O-glucuronidation | N-glucuronidation |
| --- | --- | --- | --- | --- | --- | --- | --- | --- |
| 1 |  | 8(-0.218) |  |  |  | 15(-0.969) | 16(-0.856) |  |
| 2 |  | 10(-0.351) |  |  |  | 16(-0.970) | 14(-0.830) |  |
| 3 |  | 13(-0.783) |  |  |  | 13(-0.929) | 2(-0.028) |  |
| 4 |  | 17(-0.961) |  |  |  | 1(0.890) | 17(-0.873) |  |
| 5 |  | 9(-0.221) |  |  |  | 10(-0.889) | 7(-0.639) |  |
| 6 |  | 18(-0.969) |  |  |  | 13(-0.929) | 18(-0.884) |  |
| 7 |  | 15(-0.867) |  |  |  | 14(-0.931) | 12(-0.735) |  |
| 8 |  | 16(-0.932) |  |  |  | 7(-0.801) | 15(-0.853) |  |
| 9 |  | 12(-0.764) |  |  |  | 6(-0.787) | 1(0.809) |  |
| 10 |  | 14(-0.825) |  |  |  | 3(-0.646) | 13(-0.825) |  |
| 11 |  | 3(0.485) |  |  |  | 8(-0.822) | 5(-0.518) |  |
| 12 |  | 4(0.471) |  |  |  | 12(-0.920) | 11(-0.717) |  |
| 13 |  | 7(0.130) |  |  |  | 11(-0.896) | 10(-0.699) |  |
| 14 |  | 2(0.509) |  |  |  | 11(-0.896) | 9(-0.676) |  |
| 15 |  | 6(0.420) |  |  |  | 9(-0.878) | 8(-0.663) |  |
| 16 |  | 11(-0.503) |  |  |  | 2(-0.457) | 6(-0.562) |  |
| 17 |  | 11(-0.503) |  |  |  | 2(-0.457) | 6(-0.562) |  |
| 18 |  | 5(0.452) |  |  |  | 5(-0.786) | 3(-0.447) |  |
| 19 |  | 1(0.525) |  |  |  | 4(-0.763) | 4(-0.461) |  |

The rank of the prediction is presented in the column.

DeltaP = Pa-Pi is presented in the brackets; Pa is the probability that this atom would be the reacting atom and Pi is the probability that it would not be the reacting atom of the specified reaction of biotransformation.

The real reacting atoms are highlighted.


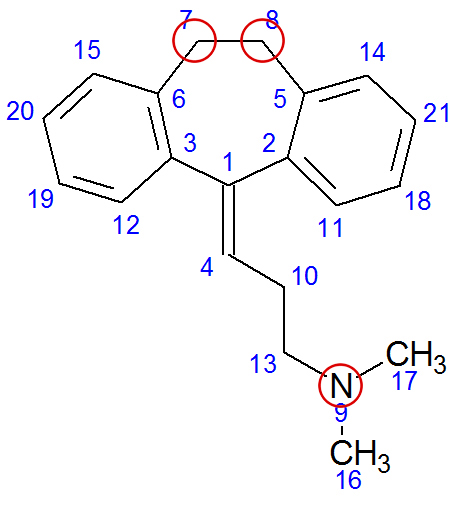


Figure 18. Structure of amitriptyline.

Table 18-1. Result of biotransformation class prediction for amitriptyline

| Pa | Pi | Reaction |
| --- | --- | --- |
| 0.830 | 0.007 | N-Dealkylation |
| 0.659 | 0.019 | N-Glucuronidation |
| 0.645 | 0.065 | Aromatic Hydroxylation |
| 0.438 | 0.077 | N-Oxidation |
| 0.237 | 0.331 | C-Oxidation |
| 0.035 | 0.734 | Aliphatic Hydroxylation |

Pa is the probability that this substance would undergo this reaction of biotransformation

Pi is the probability that this substance would not undergo this reaction of biotransformation.

The biotransformation reactions, that are experimentally observed, are highlighted

Table 18-2. Result of reacting atom prediction for amitriptyline.

| Atom number | Aliphatic  hydroxylation | Aromatic  hydroxylation | C-oxidation | N-oxidation | O-dealkylation | N-dealkylation | O-glucuronidation | N-glucuronidation |
| --- | --- | --- | --- | --- | --- | --- | --- | --- |
| 1 | 6(-0.367) |  |  |  |  | 11(-0.988) |  |  |
| 2 | 12(-0.931) |  |  |  |  | 12(-0.991) |  |  |
| 3 | 12(-0.931) |  |  |  |  | 12(-0.991) |  |  |
| 4 | 1(0.771) |  |  |  |  | 9(-0.981) |  |  |
| 5 | 8(-0.751) |  |  |  |  | 10(-0.986) |  |  |
| 6 | 8(-0.751) |  |  |  |  | 10(-0.986) |  |  |
| 7 | 2(0.716) |  |  |  |  | 7(-0.975) |  |  |
| 8 | 2(0.716) |  |  |  |  | 7(-0.975) |  |  |
| 9 | 13(-0.987) |  |  |  |  | 1(0.968) |  |  |
| 10 | 3(0.203) |  |  |  |  | 8(-0.979) |  |  |
| 11 | 9(-0.755) |  |  |  |  | 6(-0.967) |  |  |
| 12 | 9(-0.755) |  |  |  |  | 6(-0.967) |  |  |
| 13 | 4(0.019) |  |  |  |  | 3(-0.887) |  |  |
| 14 | 11(-0.882) |  |  |  |  | 5(-0.947) |  |  |
| 15 | 11(-0.882) |  |  |  |  | 5(-0.947) |  |  |
| 16 | 5(-0.073) |  |  |  |  | 2(-0.696) |  |  |
| 17 | 5(-0.073) |  |  |  |  | 2(-0.696) |  |  |
| 18 | 10(-0.818) |  |  |  |  | 4(-0.909) |  |  |
| 19 | 10(-0.818) |  |  |  |  | 4(-0.909) |  |  |
| 20 | 7(-0.713) |  |  |  |  | 4(-0.909) |  |  |
| 21 | 7(-0.713) |  |  |  |  | 4(-0.909) |  |  |

The rank of the prediction is presented in the column.

DeltaP = Pa-Pi is presented in the brackets; Pa is the probability that this atom would be the reacting atom and Pi is the probability that it would not be the reacting atom of the specified reaction of biotransformation.

The real reacting atoms are highlighted.


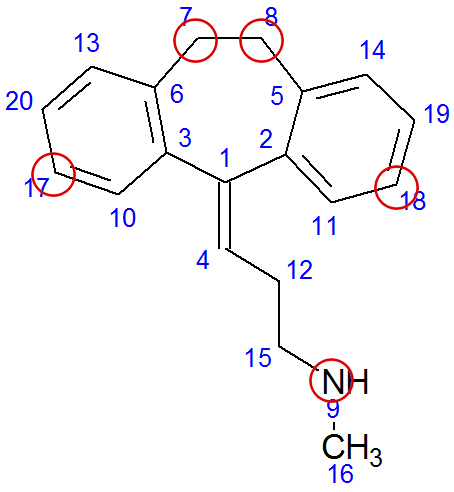


Figure 19. Structure of nortriptyline.

Table 19-1. Result of biotransformation class prediction for amitriptyline

| Pa | Pi | Reaction |
| --- | --- | --- |
| 0.697 | 0.016 | N-Dealkylation |
| 0.701 | 0.045 | Aromatic Hydroxylation |
| 0.494 | 0.040 | N-Glucuronidation |
| 0.263 | 0.204 | N-Oxidation |
| 0.180 | 0.479 | C-Oxidation |
| 0.086 | 0.561 | Aliphatic Hydroxylation |

Pa is the probability that this substance would undergo this reaction of biotransformation

Pi is the probability that this substance would not undergo this reaction of biotransformation.

The biotransformation reactions, that are experimentally observed, are highlighted

Table 19-2. Result of reacting atom prediction for nortriptyline.

| Atom number | Aliphatic  hydroxylation | Aromatic  hydroxylation | C-oxidation | N-oxidation | O-dealkylation | N-dealkylation | O-glucuronidation | N-glucuronidation |
| --- | --- | --- | --- | --- | --- | --- | --- | --- |
| 1 | 6(-0.164) | 7(-0.537) |  |  |  | 9(-0.980) |  | 7(-0.874) |
| 2 | 12(-0.807) | 6(-0.441) |  |  |  | 10(-0.988) |  | 11(-0.918) |
| 3 | 12(-0.807) | 6(-0.441) |  |  |  | 10(-0.988) |  | 11(-0.918) |
| 4 | 2(0.533) | 9(-0.651) |  |  |  | 8(-0.970) |  | 10(-0.903) |
| 5 | 9(-0.498) | 8(-0.577) |  |  |  | 9(-0.980) |  | 12(-0.919) |
| 6 | 9(-0.498) | 8(-0.577) |  |  |  | 9(-0.980) |  | 12(-0.919) |
| 7 | 1(0.740) | 5(-0.385) |  |  |  | 7(-0.964) |  | 9(-0.890) |
| 8 | 1(0.740) | 5(-0.385) |  |  |  | 7(-0.964) |  | 9(-0.890) |
| 9 | 13(-0.992) | 13(-0.983) |  |  |  | 1(0.965) |  | 1(0.955) |
| 10 | 8(-0.494) | 4(0.319) |  |  |  | 6(-0.955) |  | 6(-0.835) |
| 11 | 8(-0.494) | 4(0.319) |  |  |  | 6(-0.955) |  | 6(-0.835) |
| 12 | 5(-0.085) | 11(-0.897) |  |  |  | 8(-0.970) |  | 8(-0.881) |
| 13 | 11(-0.698) | 3(0.487) |  |  |  | 5(-0.932) |  | 4(-0.789) |
| 14 | 11(-0.698) | 3(0.487) |  |  |  | 5(-0.932) |  | 4(-0.789) |
| 15 | 4(0.087) | 12(-0.968) |  |  |  | 4(-0.906) |  | 5(-0.824) |
| 16 | 3(0.412) | 10(-0.698) |  |  |  | 2(-0.564) |  | 2(-0.346) |
| 17 | 10(-0.568) | 2(0.582) |  |  |  | 3(-0.891) |  | 3(-0.677) |
| 18 | 10(-0.568) | 2(0.582) |  |  |  | 3(-0.891) |  | 3(-0.677) |
| 19 | 7(-0.438) | 1(0.782) |  |  |  | 3(-0.891) |  | 3(-0.677) |
| 20 | 7(-0.438) | 1(0.782) |  |  |  | 3(-0.891) |  | 3(-0.677) |

The rank of the prediction is presented in the column.

DeltaP = Pa-Pi is presented in the brackets; Pa is the probability that this atom would be the reacting atom and Pi is the probability that it would not be the reacting atom of the specified reaction of biotransformation.

The real reacting atoms are highlighted.


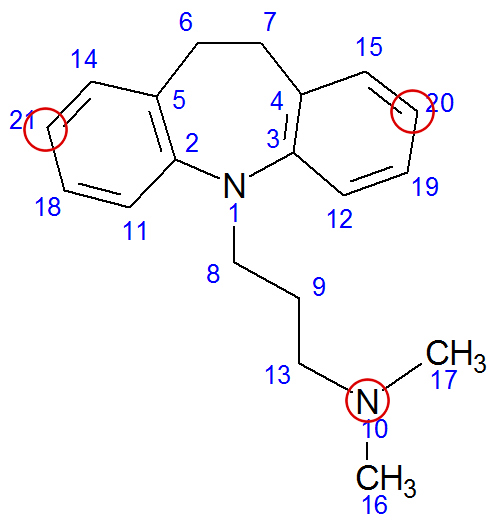


Figure 20. Structure of imipramine.

Table 20-1. Result of biotransformation class prediction for imipramine

| Pa | Pi | Reaction |
| --- | --- | --- |
| 0.887 | 0.005 | N-Dealkylation |
| 0.650 | 0.063 | Aromatic Hydroxylation |
| 0.416 | 0.052 | N-Glucuronidation |
| 0.348 | 0.135 | N-Oxidation |
| 0.290 | 0.227 | C-Oxidation |
| 0.114 | 0.495 | Aliphatic Hydroxylation |

Pa is the probability that this substance would undergo this reaction of biotransformation

Pi is the probability that this substance would not undergo this reaction of biotransformation.

The biotransformation reactions, that are experimentally observed, are highlighted

Table 20-2. Result of reacting atom prediction for imipramine.

| Atom number | Aliphatic  hydroxylation | Aromatic  hydroxylation | C-oxidation | N-oxidation | O-dealkylation | N-dealkylation | O-glucuronidation | N-glucuronidation |
| --- | --- | --- | --- | --- | --- | --- | --- | --- |
| 1 |  | 13(-0.968) |  |  |  | 2(0.075) |  | 2(0.338) |
| 2 |  | 6(-0.661) |  |  |  | 12(-0.989) |  | 12(-0.930) |
| 3 |  | 6(-0.661) |  |  |  | 12(-0.989) |  | 12(-0.930) |
| 4 |  | 8(-0.779) |  |  |  | 11(-0.988) |  | 11(-0.927) |
| 5 |  | 8(-0.779) |  |  |  | 11(-0.988) |  | 11(-0.927) |
| 6 |  | 5(-0.514) |  |  |  | 10(-0.981) |  | 10(-0.907) |
| 7 |  | 5(-0.514) |  |  |  | 10(-0.981) |  | 10(-0.907) |
| 8 |  | 10(-0.952) |  |  |  | 8(-0.961) |  | 9(-0.870) |
| 9 |  | 12(-0.967) |  |  |  | 6(-0.955) |  | 7(-0.827) |
| 10 |  | 11(-0.961) |  |  |  | 1(0.910) |  | 1(0.903) |
| 11 |  | 3(0.217) |  |  |  | 9(-0.972) |  | 8(-0.848) |
| 12 |  | 3(0.217) |  |  |  | 9(-0.972) |  | 8(-0.848) |
| 13 |  | 9(-0.949) |  |  |  | 4(-0.875) |  | 4(-0.671) |
| 14 |  | 4(0.097) |  |  |  | 7(-0.956) |  | 6(-0.811) |
| 15 |  | 4(0.097) |  |  |  | 7(-0.956) |  | 6(-0.811) |
| 16 |  | 7(-0.697) |  |  |  | 3(-0.776) |  | 3(-0.362) |
| 17 |  | 7(-0.697) |  |  |  | 3(-0.776) |  | 3(-0.362) |
| 18 |  | 2(0.665) |  |  |  | 5(-0.928) |  | 5(-0.718) |
| 19 |  | 2(0.665) |  |  |  | 5(-0.928) |  | 5(-0.718) |
| 20 |  | 1(0.821) |  |  |  | 5(-0.928) |  | 5(-0.718) |
| 21 |  | 1(0.821) |  |  |  | 5(-0.928) |  | 5(-0.718) |

The rank of the prediction is presented in the column.

DeltaP = Pa-Pi is presented in the brackets; Pa is the probability that this atom would be the reacting atom and Pi is the probability that it would not be the reacting atom of the specified reaction of biotransformation.

The real reacting atoms are highlighted.


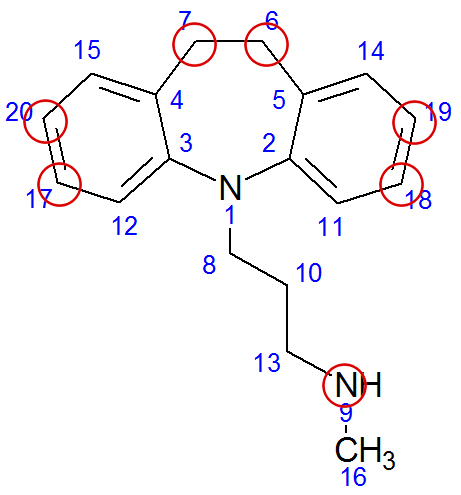


Figure 21. Structure of desipramine.

Table 21-1. Result of biotransformation class prediction for desipramine

| Pa | Pi | Reaction |
| --- | --- | --- |
| 0.826 | 0.007 | N-Dealkylation |
| 0.697 | 0.047 | Aromatic Hydroxylation |
| 0.322 | 0.090 | N-Glucuronidation |
| 0.239 | 0.327 | C-Oxidation |
| 0.179 | 0.284 | N-Oxidation |
| 0.189 | 0.348 | Aliphatic Hydroxylation |

Pa is the probability that this substance would undergo this reaction of biotransformation

Pi is the probability that this substance would not undergo this reaction of biotransformation.

The biotransformation reactions, that are experimentally observed, are highlighted

Table 21-2. Result of reacting atom prediction for desipramine.

| Atom number | Aliphatic  hydroxylation | Aromatic  hydroxylation | C-oxidation | N-oxidation | O-dealkylation | N-dealkylation | O-glucuronidation | N-glucuronidation |
| --- | --- | --- | --- | --- | --- | --- | --- | --- |
| 1 | 12(-0.953) | 11(-0.933) |  |  |  | 2(0.061) |  |  |
| 2 | 11(-0.879) | 6(-0.548) |  |  |  | 12(-0.985) |  |  |
| 3 | 11(-0.879) | 6(-0.548) |  |  |  | 12(-0.985) |  |  |
| 4 | 10(-0.686) | 7(-0.651) |  |  |  | 11(-0.980) |  |  |
| 5 | 10(-0.686) | 7(-0.651) |  |  |  | 11(-0.980) |  |  |
| 6 | 1(0.632) | 5(-0.386) |  |  |  | 10(-0.970) |  |  |
| 7 | 1(0.632) | 5(-0.386) |  |  |  | 10(-0.970) |  |  |
| 8 | 3(0.331) | 9(-0.905) |  |  |  | 8(-0.948) |  |  |
| 9 | 13(-0.973) | 13(-0.978) |  |  |  | 1(0.910) |  |  |
| 10 | 5(-0.086) | 10(-0.926) |  |  |  | 6(-0.937) |  |  |
| 11 | 9(-0.684) | 3(0.243) |  |  |  | 9(-0.959) |  |  |
| 12 | 9(-0.684) | 3(0.243) |  |  |  | 9(-0.959) |  |  |
| 13 | 4(0.166) | 12(-0.957) |  |  |  | 4(-0.895) |  |  |
| 14 | 6(-0.383) | 4(0.130) |  |  |  | 7(-0.942) |  |  |
| 15 | 6(-0.383) | 4(0.130) |  |  |  | 7(-0.942) |  |  |
| 16 | 2(0.386) | 8(-0.746) |  |  |  | 3(-0.682) |  |  |
| 17 | 8(-0.452) | 2(0.651) |  |  |  | 5(-0.908) |  |  |
| 18 | 8(-0.452) | 2(0.651) |  |  |  | 5(-0.908) |  |  |
| 19 | 7(-0.430) | 1(0.803) |  |  |  | 5(-0.908) |  |  |
| 20 | 7(-0.430) | 1(0.803) |  |  |  | 5(-0.908) |  |  |

The rank of the prediction is presented in the column.

DeltaP = Pa-Pi is presented in the brackets; Pa is the probability that this atom would be the reacting atom and Pi is the probability that it would not be the reacting atom of the specified reaction of biotransformation.

The real reacting atoms are highlighted.


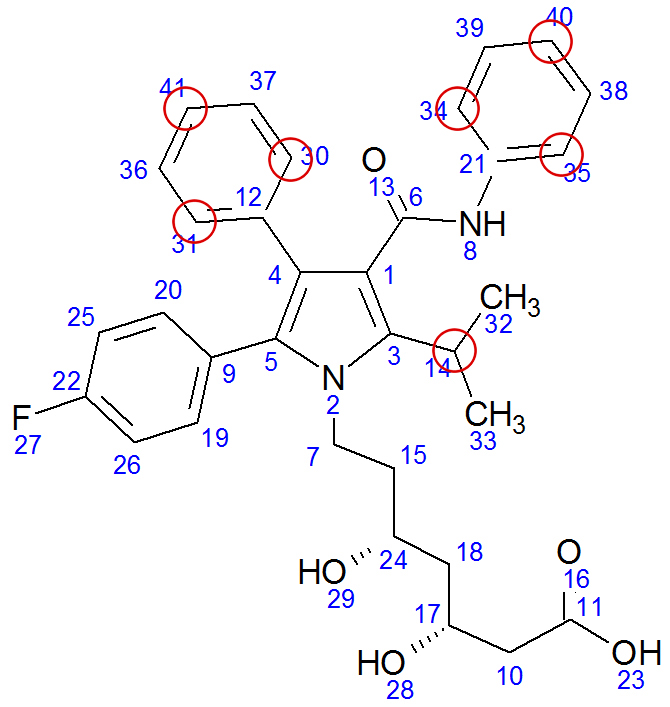


Figure 22. Structure of atorvastatin.

Table 22-1. Result of biotransformation class prediction for atorvastatin

| Pa | Pi | Reaction |
| --- | --- | --- |
| 0.708 | 0.043 | Aromatic Hydroxylation |
| 0.467 | 0.063 | O-Glucuronidation |
| 0.167 | 0.298 | N-Oxidation |
| 0.198 | 0.332 | Aliphatic Hydroxylation |
| 0.053 | 0.315 | N-Dealkylation |
| 0.066 | 0.491 | N-Glucuronidation |
| 0.147 | 0.585 | C-Oxidation |
| 0.053 | 0.512 | O-Dealkylation |

Pa is the probability that this substance would undergo this reaction of biotransformation

Pi is the probability that this substance would not undergo this reaction of biotransformation.

The biotransformation reactions, that are experimentally observed, are highlighted

Table 22-2. Result of reacting atom prediction for atorvastatin.

| Atom number | Aliphatic  hydroxylation | Aromatic  hydroxylation | C-oxidation | N-oxidation | O-dealkylation | N-dealkylation | O-glucuronidation | N-glucuronidation |
| --- | --- | --- | --- | --- | --- | --- | --- | --- |
| 1 | 14(-0.310) | 27(-0.724) |  |  |  | 33(-0.760) |  |  |
| 2 | 28(-0.549) | 30(-0.766) |  |  |  | 7(0.036) |  |  |
| 3 | 18(-0.350) | 32(-0.826) |  |  |  | 34(-0.801) |  |  |
| 4 | 11(-0.297) | 17(-0.505) |  |  |  | 31(-0.623) |  |  |
| 5 | 17(-0.333) | 23(-0.618) |  |  |  | 32(-0.657) |  |  |
| 6 | 6(-0.063) | 31(-0.772) |  |  |  | 30(-0.557) |  |  |
| 7 | 2(0.073) | 22(-0.581) |  |  |  | 21(-0.361) |  |  |
| 8 | 34(-0.765) | 31(-0.772) |  |  |  | 4(0.168) |  |  |
| 9 | 22(-0.429) | 10(-0.295) |  |  |  | 28(-0.419) |  |  |
| 10 | 9(-0.254) | 29(-0.748) |  |  |  | 26(-0.413) |  |  |
| 11 | 32(-0.586) | 28(-0.735) |  |  |  | 19(-0.337) |  |  |
| 12 | 21(-0.377) | 12(-0.329) |  |  |  | 27(-0.414) |  |  |
| 13 | 12(-0.299) | 14(-0.410) |  |  |  | 17(-0.310) |  |  |
| 14 | 1(0.639) | 19(-0.527) |  |  |  | 1(0.571) |  |  |
| 15 | 5(-0.026) | 25(-0.642) |  |  |  | 18(-0.316) |  |  |
| 16 | 26(-0.470) | 16(-0.457) |  |  |  | 16(-0.243) |  |  |
| 17 | 25(-0.465) | 26(-0.709) |  |  |  | 23(-0.392) |  |  |
| 18 | 4(-0.025) | 18(-0.509) |  |  |  | 13(-0.216) |  |  |
| 19 | 13(-0.309) | 8(-0.049) |  |  |  | 11(-0.065) |  |  |
| 20 | 13(-0.309) | 8(-0.049) |  |  |  | 11(-0.065) |  |  |
| 21 | 29(-0.556) | 13(-0.396) |  |  |  | 29(-0.434) |  |  |
| 22 | 24(-0.457) | 9(-0.230) |  |  |  | 14(-0.231) |  |  |
| 23 | 33(-0.640) | 21(-0.579) |  |  |  | 24(-0.397) |  |  |
| 24 | 10(-0.259) | 24(-0.639) |  |  |  | 20(-0.346) |  |  |
| 25 | 31(-0.580) | 2(0.115) |  |  |  | 9(0.017) |  |  |
| 26 | 31(-0.580) | 2(0.115) |  |  |  | 9(0.017) |  |  |
| 27 | 15(-0.318) | 11(-0.315) |  |  |  | 15(-0.240) |  |  |
| 28 | 30(-0.566) | 20(-0.573) |  |  |  | 22(-0.380) |  |  |
| 29 | 27(-0.488) | 17(-0.505) |  |  |  | 25(-0.399) |  |  |
| 30 | 20(-0.372) | 7(-0.015) |  |  |  | 8(0.018) |  |  |
| 31 | 20(-0.372) | 7(-0.015) |  |  |  | 8(0.018) |  |  |
| 32 | 3(0.062) | 15(-0.456) |  |  |  | 12(-0.113) |  |  |
| 33 | 3(0.062) | 15(-0.456) |  |  |  | 12(-0.113) |  |  |
| 34 | 23(-0.448) | 6(0.009) |  |  |  | 10(-0.019) |  |  |
| 35 | 23(-0.448) | 6(0.009) |  |  |  | 10(-0.019) |  |  |
| 36 | 16(-0.328) | 3(0.107) |  |  |  | 5(0.155) |  |  |
| 37 | 16(-0.328) | 3(0.107) |  |  |  | 5(0.155) |  |  |
| 38 | 19(-0.354) | 5(0.027) |  |  |  | 6(0.074) |  |  |
| 39 | 19(-0.354) | 5(0.027) |  |  |  | 6(0.074) |  |  |
| 40 | 8(-0.237) | 1(0.125) |  |  |  | 2(0.201) |  |  |
| 41 | 7(-0.218) | 4(0.088) |  |  |  | 3(0.181) |  |  |

The rank of the prediction is presented in the column.

DeltaP = Pa-Pi is presented in the brackets; Pa is the probability that this atom would be the reacting atom and Pi is the probability that it would not be the reacting atom of the specified reaction of biotransformation.

The real reacting atoms are highlighted.
